# Dynamic maternal synthesis and segregation of the germ plasm organizer, Bucky ball, in chicken oocytes and follicles

**DOI:** 10.1038/s41598-024-78544-7

**Published:** 2024-11-12

**Authors:** Sabine Klein, Roland Dosch, Sven Reiche, Wilfried A. Kues

**Affiliations:** 1 Friedrich-Loeffler-Institut, Institute of Farm Animal Genetics, Department of Biotechnology, Stem Cell Unit, Mariensee, Höltystr. 10, 31535 Neustadt, Germany; 2 Institut Für Humangenetik, Department of Developmental Biochemistry, Georg-August-Universität Göttingen, 37077 Göttingen, Germany; 3 Dept. of Experimental Animal Facilities and Biorisk Management, Friedrich-Loeffler-Institut, Südufer 10, 17493 Greifswald - Insel Riems, Germany

**Keywords:** Germline specification, Oocytes, Folliculogenesis, Balbiani body, Germ plasm, Chicken, Germline development, Stem cells, Cell biology, Stem cells

## Abstract

Maternal germ plasm determines the germline in birds. Previously, we proposed the chicken-specific Bucky ball (cBuc) as a functional equivalent of the *zebrafish* germ plasm organizer. This study demonstrated the maternal cBuc synthesis, and verified a highly dynamic distribution of Bucky ball from oocyte nests to maturing follicles using specific antibodies. The dynamic re-localization of cBuc from the ovarian stroma to the granulosa cells, and the Balbiani structure of the oocyte was revealed. Following the accumulation of cBuc in the Balbiani body, an increased signal of chicken vasa homolog (CVH) in close contact to cBuc could be detected. Highest transcription of cBuc was recorded in follicles with diameters up to 500 µm. First RNA-interference experiments in an in-vivo follicle culture assay revealed inhibiting effects on cBuc in small follicles. These data demonstrate the maternal origin of cBuc, and underpin its role as germ plasm organizer.

## Introduction

Specification of the germline cell lineage is crucial for the survival and evolved independently in different species principally based on two main strategies. One is the local induction of germline specification in pluripotent stem cells by the surrounding somatic cells. In this case, primordial germ cells are segregated from a previously undifferentiated embryonic stem cell population, as it is known for example from mammalian species including humans^1–3^, and also in urodeles^2,4–6^.

The second strategy is based on the inheritance of maternally provided germ plasm as basis for germline specification, and is found in species with very rapid early embryonic cell divisions. Astonishingly, this strategy evolved independently for very different species such as *Drosophila, C. elegans,* zebrafish (*Danio rerio*), and *Xenopus* ^6,7^. The maternally arranged germ plasm and the delivery to a limited, and species-specific number of cells in the early cleavage embryo are prerequisites for germline development. The unequal segregation of germline specific proteins, RNAs and maternal cell organelles compacted within the so called ‘germ plasm’ initiate the first cell-lineage specification^1–4^ during the earliest phase of embryogenesis and define the germline already before blastula formation. For this purpose, the germ plasm components are condensed in a membrane-less intracytoplasmic compartment, the so called Balbiani body, which is described for zebrafish^2,8^, mouse^9^ *Drosophila*^10^ and many other species in early stage oocytes^11^. Prominent germline-specific RNAs and proteins engulfed into the germ plasm from insects to vertebrates are homologs for NANOS, Pumilio, VASA, Deleted In Azoospermia Like (DAZL), Piwi, Dead end (DND), and Tudordomain proteins (TDRDn)^12–16^. However, there is a still ongoing debate, which of the two principal mechanisms is functional in birds^2,17^.

The firstly described germ plasm organizer, the riboprotein called oskar from *Drosophila*^18,19^, proved to show impressive functional homologies to the later discovered vertebrate protein called Bucky ball in zebrafish^7^. The name ‘Bucky ball’ was chosen to illustrate a major characteristic of this protein, the separation of components important for germline specification within the cytoplasm in a condensed area without a membrane, in the so-called germ plasm. The most condensed state of the germ plasm is called Balbiani body and found in early oocytes^8,20^. However, this high degree of condensation in a cytoplasmic area near the nucleus is a transient state of the germ plasm in young follicular stages. During further growth of the follicles, a dynamic shift of the germ plasm to a coronal layer underneath the vitelline membrane is observed in zebrafish^1^, and also in chicken (*Gallus gallus*)^21^. The liquid–liquid phase separation (LLPS) is an inherent property of the intrinsically disordered ribonucleoprotein granules of oskar in *Drosophila*^19^ and the intrinsically disordered proteins (IDPs) Bucky ball in zebrafish^22^ and Xvelo1 in *Xenopus*^23^. These IDPs are found in different stages of germline differentiation from yeast to mammals^24^, and provide the basis for their germ plasm organizer competence. The irregular folding and incomplete secondary structure of IDPs are at present the only common basis for a time restricted functional silencing^1,7,8,18,20,25–29^.

Specific interactions to several germline proteins including VASA, DAZL, NANOS, TDRD6a and others is realized via short, but highly specific binding motifs at the Bucky ball protein in zebrafish^14,30,31^.

Interestingly, the zebrafish Bucky ball has no DNA sequence-homolog in chicken (*Gallus gallus dom.*), however, a synteny-based search predicted the un-annotated reading frame of LOC420748 as potential equivalent^26^. Previously, we substantiated the encoded protein of LOC420748 as a functional chicken Bucky ball (cBuc) equivalent in chicken embryos^32^.

The description of cBuc as germ plasm organizer is a starting point for further investigations on primordial germ cell specification. There is a number of germline associated genes already identified in chicken oocytes and primordial germ cells of early embryos such as *Dead End Homologue (CDH), DAZL, NANOG, POU5F1,* chicken *vasa homolog (CVH)*^15,33–39^ and a in silico prediction of their protein interactions based on conserved DAZL-binding motifs^40^. In this context, first studies provide interesting data on quantitative expression of the different candidates involved in germline specification and intensify the possible importance of the zygotic genome activation for germline specification. However, the functional network of the different components within the intrauterine germ plasm specification remains largely elusive and warrants further experimental testing to verify the interrelationship and regulatory dependence of the different proteins and RNAs. Ghanem and Johnson investigated the follicle recruitment to the exponentially growing phase and maturation, and concluded that follicles at diameters of around 1 mm might be already recruited into the hierarchy of exponential growth and maturation^41^.

In the current study, we first generated and characterized specific monoclonal antibodies against cBuc, and employed them to investigate the dynamic localization of Bucky ball during oogenesis. The advanced synthesis of cBuc related to a specific local increase of the germline marker CVH support the importance of cBuc as germ plasm organizer for avian species.

## Results

### Generation of chicken-specific Bucky ball antibodies and analysis for antibody specificity

Previous data investigating cBuc in chicken embryos were generated with cross-reacting polyclonal antibodies directed against the full-length *zebrafish* Bucky ball (zBuc-pAB), which share only limited homology with the chicken variant^24^. To extend this study to early folliculogenesis, specific monoclonal antibodies against chicken Bucky ball were produced.

**Fig. 1:**
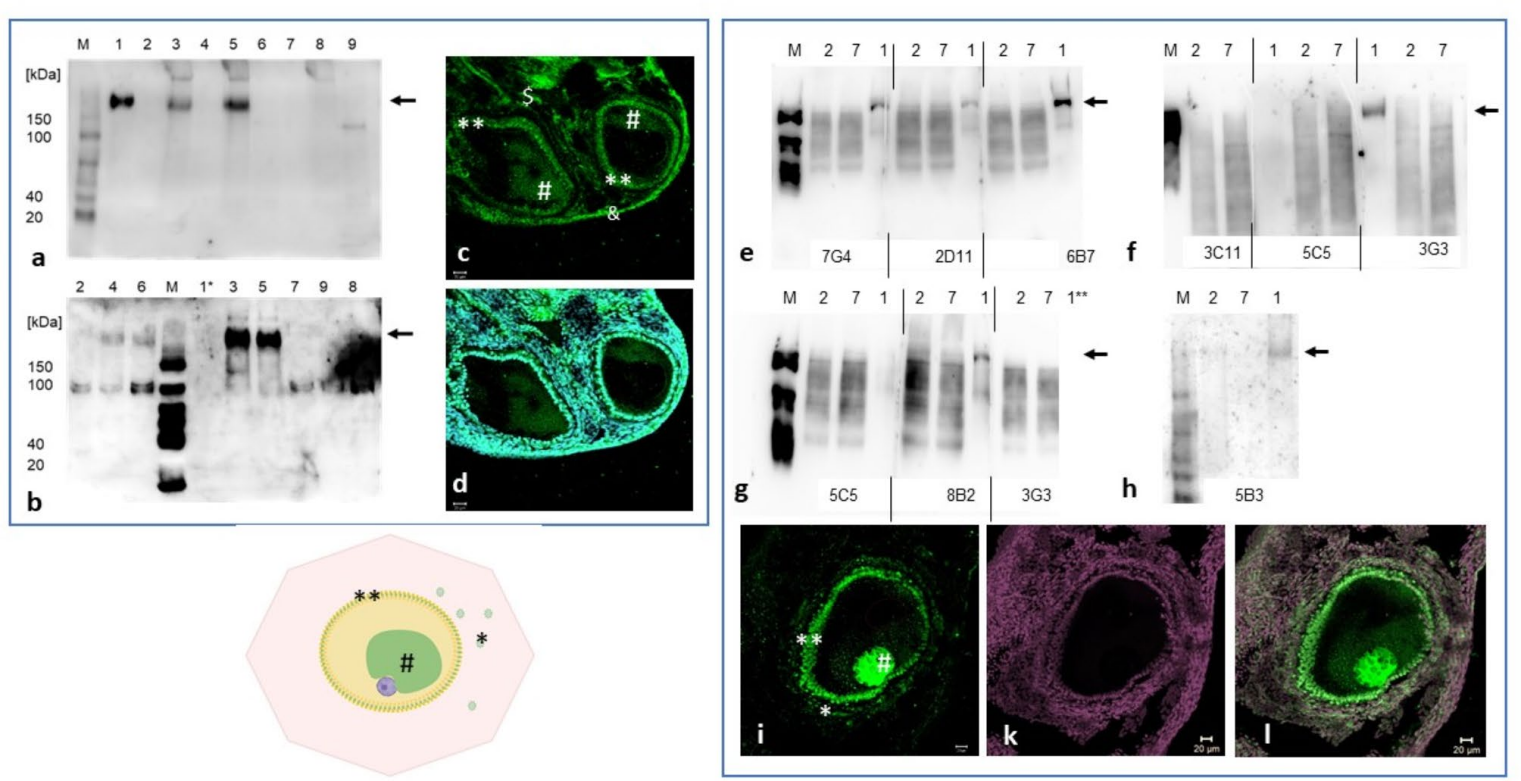
Antibody assessment of the newly raised cBuc- antibodies (**a**, **b**) Western blot testing of sera, the mice sera from animal #1080 (**a**) and #1801 (**b**) were probed against blots loaded with a protein ladder (SmoBio, #WM1000, Germany); 1,3,5 repeated pellet fractions of DF1 cells with expression of recombinant chicken Bucky ball indicated by arrows; 2, 4, 6 the corresponding soluble fractions, and 7, 8, 9 fractions of control (non-transfected cells). Note, the Bucky ball proteins concentrate in the pellet fraction and migrated much slower than expected for the calculated molecular weight, most likely reflecting the liquid–liquid phase separation properties of IDP proteins. 1*, due to an error, no sample was loaded. (**c**, **d**) Immunohistochemical labeling of two early stage III follicles with serum from mouse 1081 after 4th booster immunization. Ovarian sections (**c**) cBuc label – (green), (**d**) overlay of cBuc and DNA-stain DAPI (cyan), specific labeling of Balbiani bodies (#) and granulosa cells (**) could be demonstrated, although additional labeling of epidermal cells ($) and vasculature (&) was still apparent; (**e**, **f**) Testing of the hybridoma supernatants, the blots were redundantly loaded with soluble (2, 7) and pellet fractions (1) and cut into stripes (indicated by vertical lines), and subsequently probed individually with hybridoma supernatants. Arrows indicate specific binding of Bucky ball. 1** indicate erroneous picture processing of the original image, resulting in cropping of the last lane. (**i**, **l**) Labeling of one primary follicle (stage III—diameter of ~ 150 µm) with a mono-clonally selected hybridoma (5B3); (**i**) cBuc- green in granulosa cells (**) and Balbiani body (#); (**k**) SIR DNA stain (magenta); (**l**) overlay of cBuc and DNA stain; scales: 20 µm.

For the generation of monoclonal antibodies, a domain consisting of amino acids 155–354 of the cBuc protein was expressed in *E. coli*. The purified protein was used to immunize two mice and after the forth booster injection, the mice sera showed a specific reactivity against the full-length cBuc in Western blotting (Fig. 1 a, b) and also distinct immunoreactivity in ovarian follicles (Fig. 1c cBuc label—green, Fig. 1d overlay of cBuc and DNA counterstaining – cyan).

After the fifth booster, 768 hybridoma clones were raised as described elsewhere^42^. Among these, 10 clones showed a high reactivity in an ELISA using the recombinant protein, out of which 6 supernatants also showed a specific binding to the full-length recombinant Bucky ball^24^ expressed in chicken immortalized cells (Fig. 1 e, f, g, h).

The specificity of binding of the polyclonal cBuc serum as demonstrated in Fig. 1c could be considerably improved by selection of monoclonal hybridomas (5B3) as shown in Fig. 1i with green cBuc labeling, complemented with the DNA-counterstaining in Fig. 1k and the overlay of both in Fig. 1l, and demonstrated for all three immunoreactive hybridomas 8B2, 7G4, and 5B3 in Supplementary Figure S1, column 1 to 3. The additional labels in epithelial tissue ($) and vasculature (&) which were abundant for both sera (Fig. 1c) was completely gone with all three hybridomas.

Although the heterologous antibodies zBuc-pAB provided consistent low intensity signals for cBuc, the signal intensity was week especially for the follicles of stage III and IV as indicated by direct comparison of same stage immunoreactivity of stage III and stage IV follicles as presented in Supplementary Figure S2. The granular condensation of cBuc could only be differentiated with the cBuc antibodies especially in the Balbiani body (Supplementary Figures S2a, c), the cross-reacting zBuc-pAB did not visualize this granular structure in the Balbiani body and barely the circular arrangement of cBuc in the growing follicles (Supplementary Figure S2g).

Controls without primary antibody (Supplementary Figures S3 a-e) and isotype controls with IgG1 anti-mouse antibodies (Supplementary Figures S3 f.–h) verified the specificity of the cBuc-labeling with the applied immuno-histochemistry protocol. No labeling with the secondary antibody alone could be detected in the green channel (S-Figure S3a and S3d, green label) nor with the isotype control with the corresponding secondary antibody in the red channel (Supplementary Figure S3f.). Only the previously described unspecific signals originating from yolk ($) could be observed. The counterstaining with SIR (Supplementary Figure S3b and S3g as well as phalloidin for F-actin (cyan in Supplementary Figure S3d, combined to secondary antibody binding of the anti-mouse Alexa 555 to unspecific yolk ($ in green)) and the mitochondrial marker Mitobrillant Red (Supplementary Figure S3e (magenta) in overlay to F-actin (cyan) revealed completely independent signals.

### Immuno-histochemical localization of Bucky ball (cBuc) protein during folliculogenesis

**Fig. 2:**
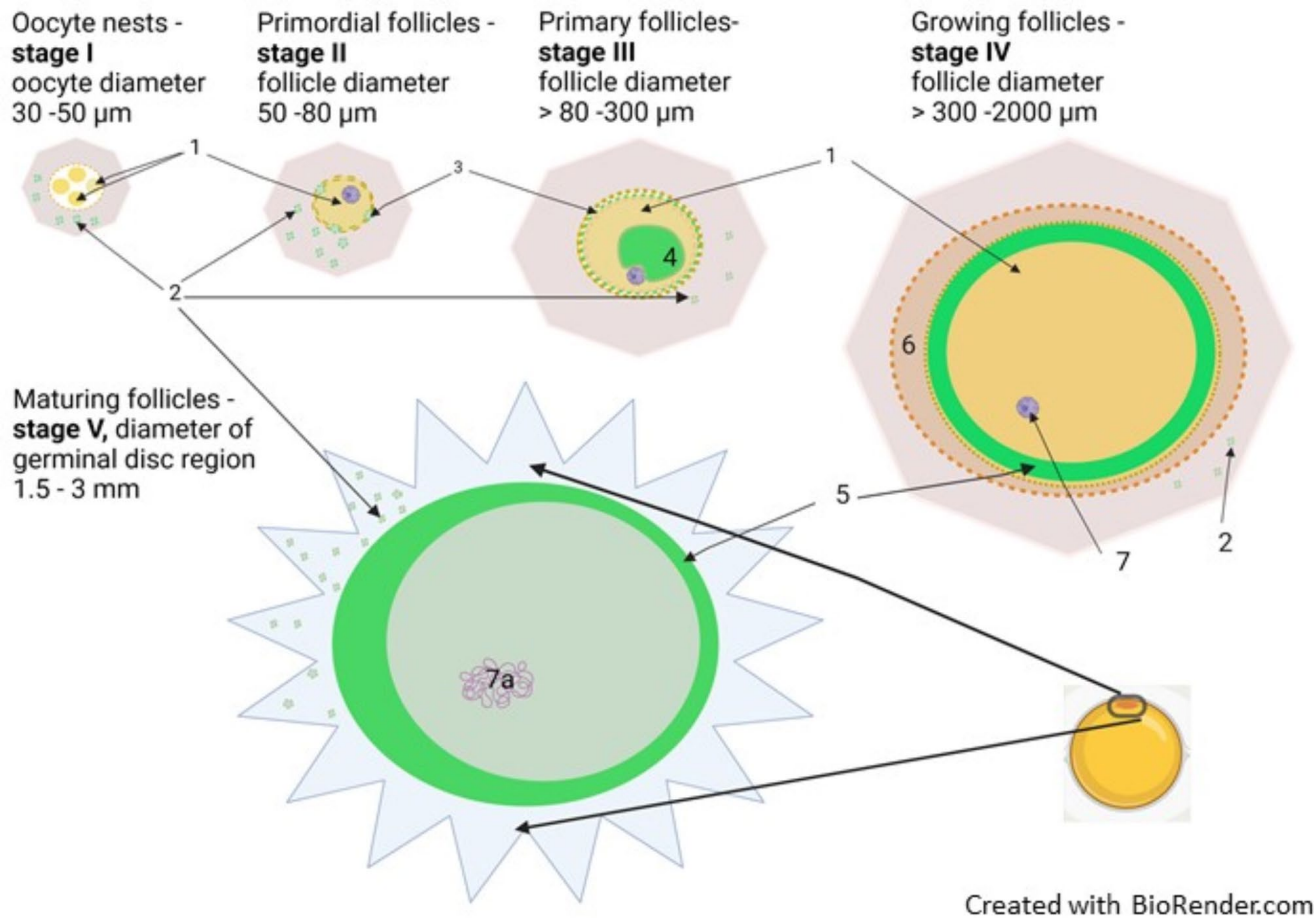
Chicken Buc dynamics during folliculogenesis. Scheme of oocyte and follicle development in adult chicken ovaries staged according to size and cellular composition, and highlighting the dynamic localization of germ plasm organizer protein Bucky ball (green). Stage I: oocyte nests in the ovarian epithelium, with oocyte diameters < 50 µm; Stage II: primordial follicles with first granulosa cells; Stage III: primary follicles with tubular arranged granulosa cells and Balbiani body, follicle diameters of  > 80 to 300 µm; Stage IV: growing follicles with decondensed Balbiani body and circular germ plasm underneath the vitelline membrane; Stage V: Germ plasm surrounds the germinal vesicle in the germinal disc region of maturing follicles before ovulation; 1, oocytes; 2, stromal cells with Bucky ball labeling; 3, granulosa cells with Bucky ball; 4, cBuc in the condensed Balbiani body; 5, circular arrangement of cBuc including germ plasm underneath the vitelline membrane after de-condensation of the Balbiani body; 6, granulosa and theca cell layer; 7, cell nucleus; and 7a, meiotic chromosomes.

**Fig. 3:**
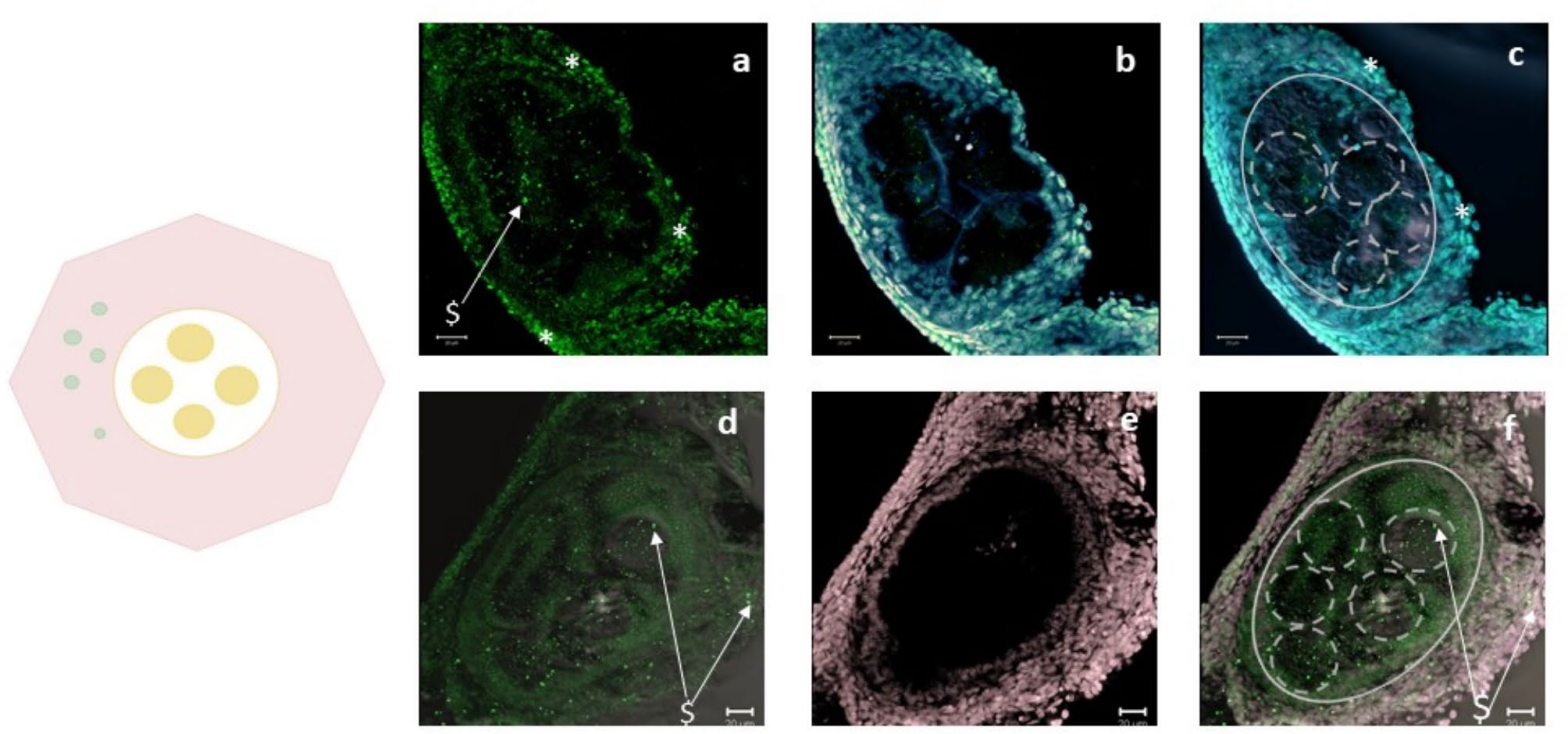
Stage I, first cBuc expression in stromal cells surrounding oocyte nests. (**a-c**) Oocyte nest with at least four oocytes (oocytes 30–50 µm); (**a**) cBuc labeled with Alexa 488 (green); (**b**) DNA stain (DAPI -cyan); and (**c**) cBuc and DNA stain; low intensity staining of cBuc in some cells of the stroma around the oocytes is indicated in a (*). (**d-f**) The control staining of an oocyte nest of minimum 5 oocytes without primary antibodies, but secondary antibodies against rabbit and mouse IgG resulted in larger and pointed brighter spots and probably originate from autofluorescence by yolk particles. The bright pointed spots from these particles ($) are seen also in (**a**), (**d–f**) should be regarded as artefacts; (**d**) cBuc channel, (**b**) DAPI (cyan), (**e**) SIR (magenta), (**c**) and (**f**) overlay of cBuc, DNA-stain and DIC contrast; scales 20 µm. Oocyte nests are indicated by grey ellipsoid outline, the recognizable oocytes by broken outlines in (**c**) and (**f**).

**Fig. 4:**
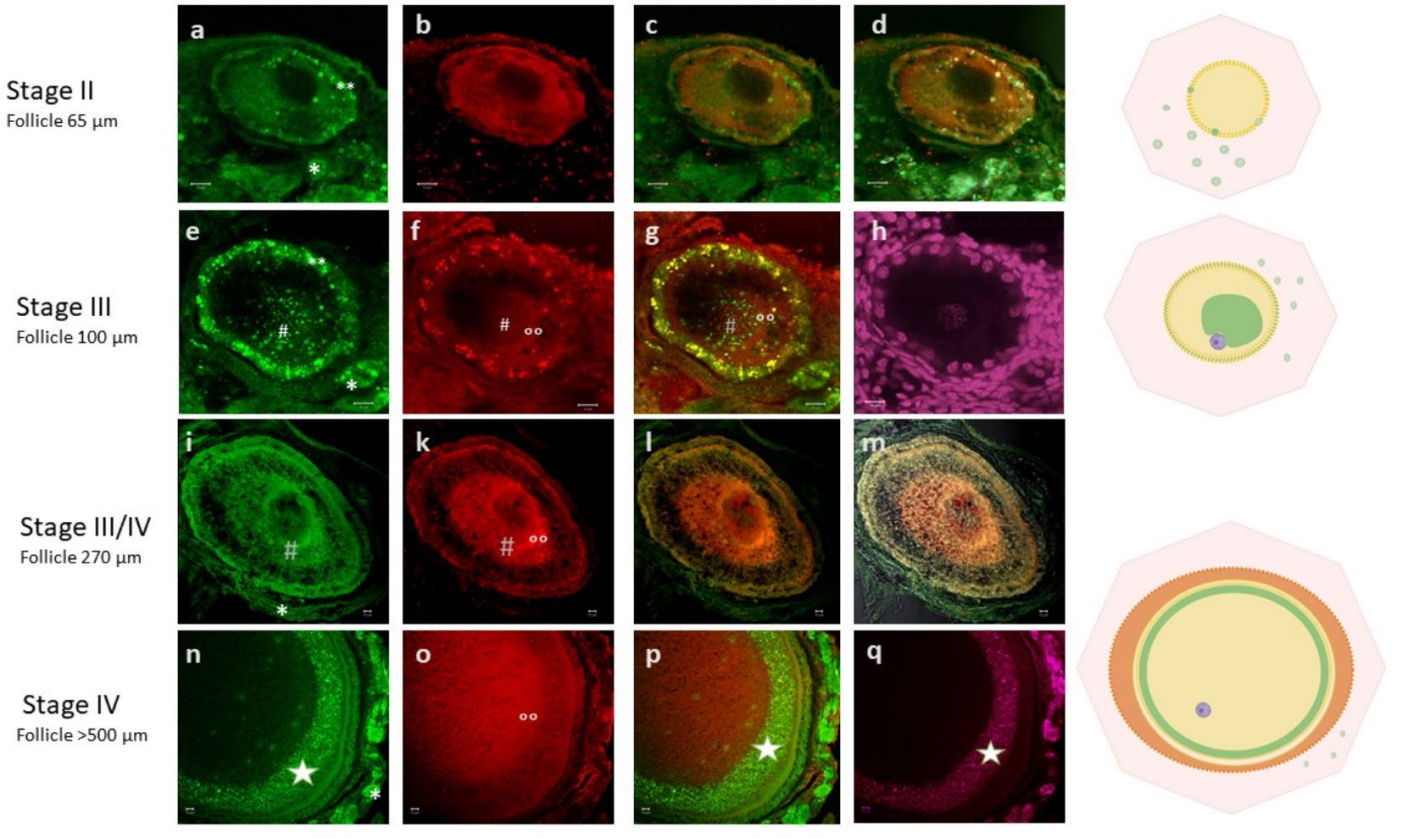
Comparative labeling for cBuc and CVH in four follicle stages. Labeling of follicles in follicular stages II to IV for cBuc in column 1 (green, **a**), (**e**), (**i**), (**n**) and CVH in column 2 (red °°, **b**), (**f**), (**k**), (**o**); column 3 (**c**), (**g**), (**l**), (**p**) visualizes the overlay of cBuc and CVH; and column 4 represents the SIR DNA label (magenta) in (**h**) and (**q**), or the overlay including the DIC contrast in (**d**) and (**m**). Highest condensation of cBuc labeling is seen in the Balbiani body (**), whereas granulosa cells (*) and the circular arranged cBuc labels underneath the vitelline membrane (*) appear more diffuse. CVH is arranged in high concentration around the Balbiani body (°°) as marked by cBuc (#, **g**, **h**). Scales 10 µm.

**Fig. 5:**
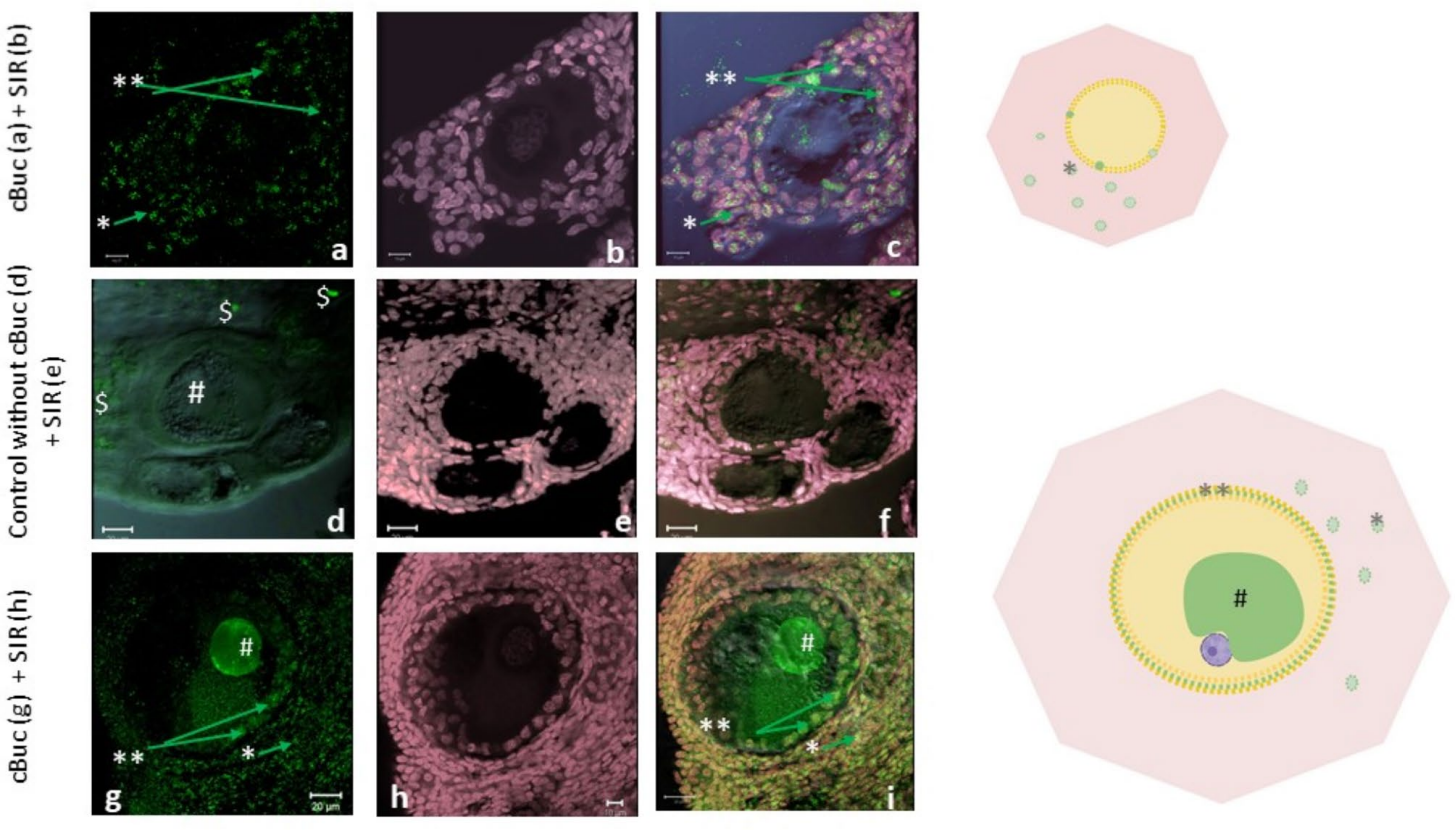
Dynamic localization of cBuc protein in follicles at stage II and III. (**a-c**) Bucky ball (**a**, green label) is first detected in the loosely arranged granulosa cells (**) in primordial follicles of stage II (50 µm oocyte diameter and onwards), indicated by SIR DNA stain in (**b**) additionally to the stromal labeling (*) in the overlay with SIR in magenta and DIC in (**c**). The Balbiani body is not yet formed. (**d-f**) Any intrafollicular labeling is not seen in controls without primary cBuc antibody. Here, only the strong and larger autofluorescence of yolk particles ($) is obvious in the channel for green fluorescence (**d**); formation of the Balbiani body (#,) is indicated in the overlay of DNA stain (SIR in (**e**), magenta) with both fluorescence channels and DIC contrast (**f**). (**g-h**) The labeling of cBuc within granulosa cells increased with follicular growth for the number of labeled cells and intensities and is released into the oocyte for accumulation into the Balbiani body. In (**a**) and (**g**) cBuc, (**b**), (**e**), and (**h**) SIR; (**c**), (**f**), (**i**) overlay of SIR, DIC and cBuc label; scales: (**a**), (**b**), (**c**) and (**h**) 10 µm; (**d-g**), (**i**) 20 µm.

**Fig. 6:**
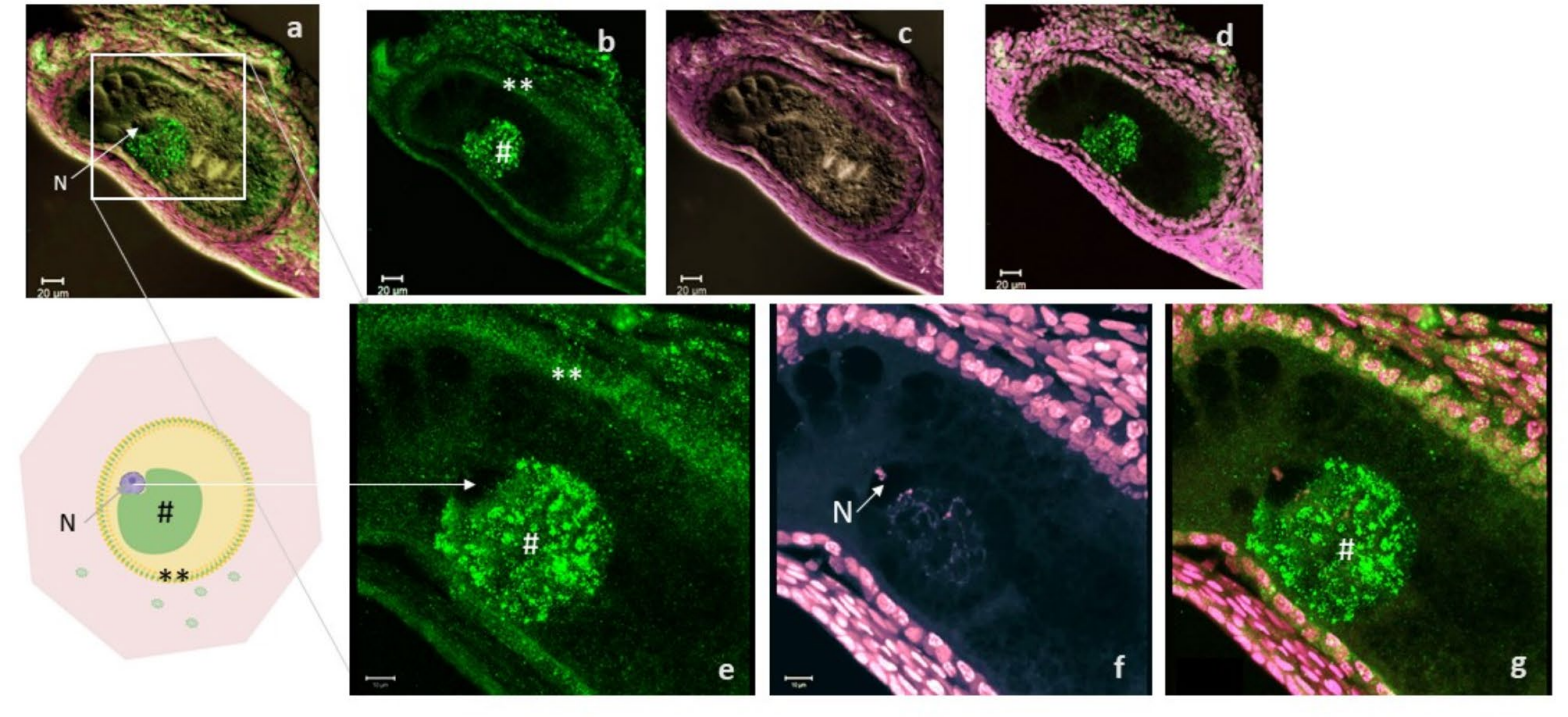
Condensed cBuc in Balbiani body aside the cell nucleus. (**a-d**) Three-channel overlay (immunostaining for cBuc (green); nuclear counterstaining with SIR (magenta) on DIC contrast); this follicle of about 150 µm diameter (**a**) was labeled with the hybridoma 7G4 antibody for cBuc (green, **b**). (**c**) represents the overlay of DIC and SIR. Figure 6d shows the overlay of cBuc and SIR of this follicle. Bucky ball is concentrated in highly granular condensates in the large Balbiani body in close proximity to the nucleus (N). (**e–g**) The high magnification frame of the follicle from (**a**) visualizes the nucleoli stained by SIR in the nucleus (**f** and **g**) in direct contact to the cBuc granules of the Balbiani body (#, **e** and **g**). The granulosa cell layer is characterized by a rather diffuse labeling for cBuc (** in **b** and **e**). [**d**, **g** = overlay of SIR – magenta and cBuc—green; **c** = cBuc, **d** + **g** = overlay cBuc + CVH; scales **a**-**d** = 20 µm, scales **e**–**g** – 10 µm].

**Fig. 7:**
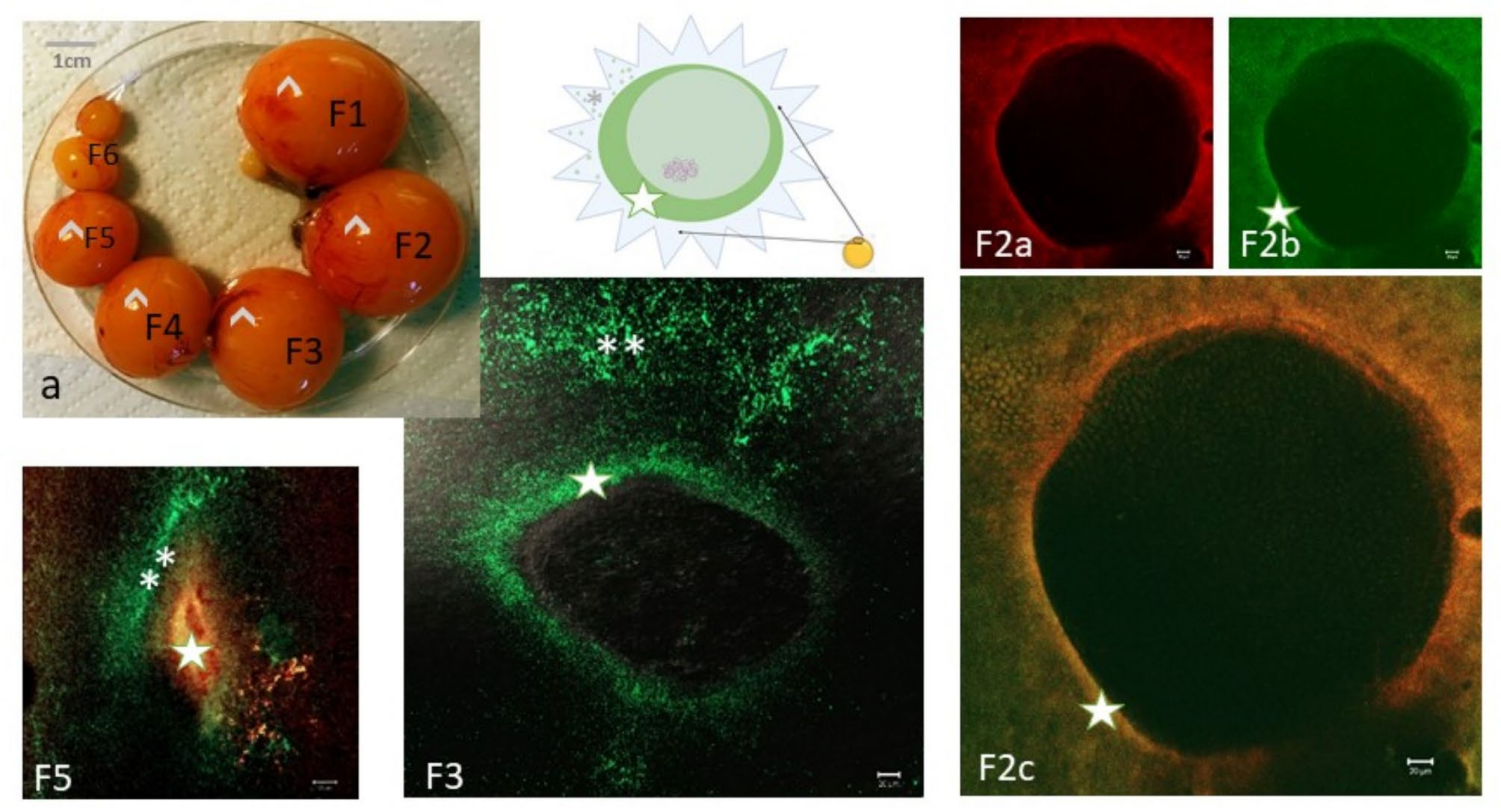
Stage V; Germinal vesicle (GV) regions of maturing chicken follicles with cBuc and CVH labeling. (**a**) At the last 7 days before ovulation, a clear hierarchy of the yolk size is recognizable. On the largest yellow follicles at the last days of follicle maturation before ovulation (F5 to F1, stage V) the GV is visible at the surface of the yolk (arrow heads in **a**). The follicle F1 will be ovulated the next day, F2 after about 48h and so on. The GV containing regions of the follicles were explanted at a diameter of around 5mm by cutting the vitelline membrane of the oocytes (the scheme above represents this germinal vesicle region with the irregular cutting edges of the surrounding vitelline membrane with adhering follicle cells. (**F5**) In the GV-region of follicle (F5 in **a**) left), the explant shows a granulosa cell population with intense cBuc label unilateral to the GV and co-labeling of cBuc (green) and CVH (red) around the GV resulting in orange color overlay. (**F3**) The F3 follicle (F3 in a) middle lower panel) is presented with the label for cBuc exclusively. The cBuc protein is shifted more to the coronal outside of the GV after considerable increase in the GV diameter. The unilateral enrichment is in near proximity to the granulosa cell population, that is rich in cBuc protein. (**F2a-c**) (**F2a**) CVH stained F2 follicle; (**F2b**) anti-zebrafish Buc serum label, (**F2c**) overlay of CVH and zBuc-pAB label; Labels for Buc (green **F5**, **F3**, **F2b**) are arranged with unilateral higher concentration (green star) around the germinal vesicle, besides an additional follicular cell population with Buc label at the same side of the follicle (**). Scales 20 µm.

In the current study, which exclusively examined follicles from adult chicken ovaries, five major stages of oocyte development could be discriminated on the basis of size (follicle diameter) and dynamic localization of cBuc: stage I: oocytes of 30–50 µm; stage II: primordial follicles of 50–80 µm; stage III: primary follicles of 80–300 µm; stage IV: growing follicles of 300–2000 µm; and stage V: germinal vesicle regions of 1500–3000 µm separated from the yolk of maturing follicles. These stages were described for their cBuc distribution in Table 1, and schematically drawn in Fig. 2. The immunolabels for cBuc (Fig. 3 stage I, stages II-IV in Fig. 4 (cBuc in a, e, l, n), Fig. 5 (cBuc in a, d, g) Fig. 6 (cBuc in a, b, d, e) and CVH (Fig. 4 b, f, k, o) and their overlays of cBuc and CVH (Fig. 4 c, g, l, p) or with SIR as third label (Fig. 4 d, h, m, q), Fig. 5and 6, respectively. The germinal vesicle regions of maturing follicles (stage V) are presented in Fig. 7 with cBuc label for F5, F3 and F2 and CVH label for F5 and F2 and Supplementary Figure S8.

**Table 1:** Stages of differential Bucky ball synthesis and deposition during folliculogenesis in chicken.

Stage	Oocyte / follicle diameter	Follicle constitution	Balbiani structure appearance	Immuno-la-beling of cBuc	Accumulation of cBuc in the Balbiani body	CVH immuno-labeling	Figures demon-strating immuno-labeling
I	Oocytes 30–50 µm	Oocytes in nests or separated oocytes with first round granulosa cells attached	No Balbiani recognized	No label in follicles, Suggested stromal cells with cBuc synthesis	Non	Non	Fig 2, stage I Fig. 3 S-Fig. S4 a-c
II	Primordial follicles 50–80 µm	Loosely arranged granulosa cells surround the oocyte	No Balbiani body recognizable yet	cBuc synthesis in granulosa cells	First fussy cBuc in oocyte cytoplasm detected	weak uniform label within the oocyte	Fig. 4 a-d, Fig. 5a-c S-Fig. S5 a-d
III	Primary follicles 80–300 µm	Tubular granulosa cell layer Theca cells	Balbiani body aggregates aside the nucleus	Intense synthesis of cBuc in granulosa cells	Unilateral transport of cBuc into the cytoplasm of oocytes for granule formation of cBuc within the clearly demarked Balbiani body	Increased labeling within the oocyte with granular condensation within the Balbiani body and increased intensity around it	Fig. 3 g-i, Fig. 4 e–f, Fig. 5d-I, Fig. 6 S-Fig. S1 S-Fig. S2 a, b e,f, S-Fig. S3, S-Fig. S6, S-Fig. S7
IV	Growing follicles > 300–2000 µm		Germ plasm inside the Balbiani structure is segregated unilateral and coronal at the inner side of the perivitelline membrane	Reduced density of cBuc in granulosa cells	wide distribution of cBuc and germ plasm proteins within the increasing follicle	CVH as weak label throughout the oocytes; Increased and condensed labeling in granules colocalizing cBuc	Fig. 4 i-q, S-Fig. S2 c, d, g, h
V	Maturing follicles during the last five days before ovulation	Superficial germinal vesicle of 1.5 to 3 mm (GV) on large yolk of 35 mm	Germ plasm concentrated at the outer rim of the GV		cBuc labeled unilateral at the outer rim of the GV #, and stroma unilateral at the GV *	CVH concentrated next to cBuc at the rim of the GV	Fig. 7 S-Fig. S8

#### Small white resting oocytes in oocyte nests and single oocytes at the beginning of follicle formation—stage I (Fig. 3 + Supplementary Figures S4a-c, S5a-d)

The first group (stage I) are small, resting oocytes in nests of the ovarian cortex as described by Guraya^43^ and Gonzalez-Moran^44^ for young chick ovaries till 35 days of age. Here, we show that these nests could be detected repeatedly in adult ovaries, too (Fig. 3). They contained 3 to 6 ovaries within one nest. The smallest oocytes had diameters of 30 to 50 µm and did not contain clear cBuc nor CVH signaling in or on the oocyte itself (Fig. 3 a; Supplementary Figure S3b, triangle), but have groups of surrounding stromal cells with weak but clear immuno-labeling for either cBuc (* in f) or CVH (° in c, g), and large dots with strong co-localizing signals ($ in Supplementary Figure S3d, h).These large dots refer to yolk particles with strong autofluorescence in green and red fluorescence ($) that is apparent in negative controls without primary antibodies, too (Fig. 3d “$”, and Supplementary Figure S3 in f, g).

#### Small white, primordial follicles (stage II)

The germ cell nests are surrounded by slowly growing follicles of the second group, which are oocytes already separated from the nests and with a successively establishing granulosa cell layer of the follicle and a size of > 50 to 80 µm diameter (Figs. 4a-d and 5a-c, Supplementary Figures S4, S5, cBuc label in green channel * and **). Granulosa cells, the first indication of folliculogenesis appear as loosely arranged, and still separated round cells. At this time, low signals for cBuc could be recorded within the granulosa cells of these primordial follicles (Figs. 3a, 5a **, Supplementary Figure S5 b, f**). In Fig. 5a, the green cBuc-labeling in first loosely arranged granulosa cells is visualized, and supplemented by SIR-counterstained DNA in Fig. 5b. The overlay is shown in Fig. 5c.

During further growth of the follicles and organization of the granulosa cell layer, these follicles are characterized by an already visible germ plasm loosely arranged in the cytoplasm and attached to the cell nucleus in the smaller follicles, before the typical circular Balbiani body could be revealed by condensation of cBuc (green) in primary follicles (Fig. 4e, i, # and yellow in the overlay with CVH (g) and SIR (h). In the Balbiani body (#), a more central and highly condensed arrangement of cBuc is found with circularly intensified CVH (°°) around it (Fig. 4f, k).

#### Primary follicles larger than 80 µm with clearly demarcated Balbiani body (stage III)

Strong specific labeling for cBuc was detected within the oocytes of follicles larger than 100 µm diameter (Fig. 4(e - h), Fig. 5d-i, Fig. 6b, d and e, g enlarged). Labeling for cBuc was not found in the nucleus of small white follicles. Figure 6 and Supplementary Figure S6, indicate impressively the close attachment of the cytoplasmic Balbiani cluster with increasing size (green condensed granulated Buc signals in Fig. 6 a, b, d, e, g, Supplementary Figure S6 a, b, c) to the smaller nucleus indicated by the two nucleoli faintly apparent in Fig. 6 e–g and Supplementary Figure S6 a, b, d magenta; and clearly identified by SIR staining and indicated by the blue circle in Supplementary Figure S6 a and d at higher magnification. Supplementary Figure S7 highlight clearly the eccentric localization of the nucleus with two condensed and intensely stained nucleoli in the left upper corner of the oocyte and the fussy RNA staining in the Balbiani body attached to the nucleus without interference of any antibody staining. In Supplementary Figure S2e and g the weaker binding of a zBuc-pAB is shown for comparison to the more intense and granular fluorescence of cBuc after staining with the monoclonal cBuc AB (Fig. 1 i, Fig. 4 e, Fig. 6 b, e, and g, Supplementary Figures S2 a, c and S6 a-c).

#### Growing follicles during initial accumulation of yellow yolk with follicle diameters above 300 µm (stage IV)

A de-condensation of cBuc and leaking from the Balbiani body, and an accompanied re-localization of cBuc circularly underneath the vitelline membrane was observed in follicles with diameters above 240 µm (Fig. 4 i - q). The highest accumulation and a cortical enrichment of cBuc was revealed predominantly at one side, while follicular size increased from 300 to 500 µm (Fig. 4 i - m). The condensed c-Buc marked Balbiani body (Fig. 4 e, green) decondenses during this time of development and is demonstrated as intermediate state in Fig. 4I to m, before the germ plasm is segregated to the circumference of the follicle successively (Fig. 4 n, follicle < 500 µm). During the initial growth of the small yellow follicles, the nucleus was shifted eccentrically by the growing Balbiani body occupying the center of the oocyte, as demonstrated in Fig. 4 e – h, before the Balbiani structure is decondensing and spreading cortically to the vitelline membrane. This fourth group of follicles resembles follicles with slowly increasing yellow yolk accumulation up to 8 mm in diameter as described^45^.

#### Germinal discs of finally maturing follicles in the last days before ovulation (stage V)

The fifth group discussed in this report are the finally maturating series of large yellow follicles. These follicles could be ordered precisely hierarchically by size of the yolk for the last 6 to 8 maturing follicles of the sequence before ovulation (Fig. 7 a, follicle arrangement). In this group of follicles, the nucleus already reached the surface of the oocyte (indicated by arrowheads in a). The germinal disc region including the nucleus as germinal vesicle (GV) and the germ plasm with the surrounding cytoplasm was removed from the huge yolk body before analysis.

These regions were explanted at a diameter of around 5 mm by cutting the vitelline membrane of the oocytes (the scheme above (Fig. 2, stage V) represents this germinal vesicle region with the irregular cutting edges of the surrounding vitelline membrane with adhering granulosa cells. After washing off the vascularized theca cell layers and the adhering yolk, the explants of the germinal vesicle region were used for immuno-histo-chemical labeling of the stage V oocytes. The labeling in the fluorescence images of Fig. 7 refers to the sequence position of the maturing follicles before ovulation as demonstrated in Fig. 7 a.

In the separated fragments of these follicles, further aggregation of Buc as well as CVH is revealed in the semi-circular Balbiani structures within the germinal vesicle region and in one condensed unilateral enriched compartment shifting the GV eccentrically (Fig. 7 F5 and Supplementary Figure S8 b-d). Around the vesicle, a highly condensed ring of CVH (red) and cBuc protein (green, star) is located (Fig. 7; F5 F3 and F2x, Supplementary Fig. 8). Outside the GV-region there are low amounts of CVH, and cBuc except patches of granulosa cells expressing either cBuc separately (*) or CVH and cBuc as visualized in Fig. 7, for the F5 and F3 follicles. The GV itself does not contain neither CVH nor cBuc in these follicles. The size of the GV at the surface of the yolk continuously increases to more than 500 µm diameter in F1-follicles until ovulation.

### Comparative labeling of cBuc and CVH from resting oocytes to maturing follicles

In the oocytes within oocyte nests neither cBuc (green, Supplementary Figure S4 b) nor CVH (red, Supplementary Figure S4 c) could be detected, although both proteins were found in separated cell groups of surrounding stromal cells of the ovary (Supplementary Figure S5 a-d, * stromal cells labeled for cBuc: ° stromal cells labeled for CVH).

Buc labeling was detected in smallest oocytes that just establish loosely arranged granulosa cells of stage II (Fig. 3 a, **). A weak cytoplasmic staining of CVH throughout the oocyte could be verified with the monoclonal antibody (HL2485, Gene tex, Germany; Fig. 4 b). However, after cBuc synthesis in the granulosa cells (*) and later in the Balbiani body (#, Fig. 4 e), with the ongoing aggregation of cBuc an considerably increased CVH labeling was visualized in these cBuc containing regions of the oocytes (Fig. 4 f).

The labeling of CVH changed from a dispersed weak appearance (Fig. 4 b) to a specific accumulation in close contact to cBuc especially at the Balbiani structure (Follicles of stage III, Fig. 4 f and k) at the beginning of transport of cBuc to the inner circumference of the follicle from a looser central arrangement of a Balbiani body in de-condensation. However, the CVH protein is arranged predominately outside and around the densely packed cBuc granules within the Balbiani body and stayed further dispersed throughout the oocyte (Fig. 4 f, k, o). Patches of stromal cells consistently labeled for cBuc during the early differentiation steps of small white follicles (Fig. 4 a, e, i, n, *). The close neighborhood of Bucky ball and CVH is kept stable, when the cBuc labeled Balbiani structure is shifted to the inner circumference of the vitelline membrane of larger growing oocytes in follicles larger than 300 µm. In Fig. 4 n – q the CVH signal is partially arranged in the same circular pattern which is located between two distinct rings of cBuc. As the inner circular cBuc label co-localizes with SIR in the overlays in Fig. 4 m and indicated by separate SIR-staining in q, we would suggest that much of the germ plasm contains mRNAs at this inner circular position that bind to cBuc.

Imaging of the growing follicles excludes most of the stroma from the field of view in imaged follicles at a size of more than 300 µm (Fig. 4 n-q) and visualize increasing intensities of cBuc and CVH labeling at unilaterally condensed areas shifted to the circumference of the cytoplasm near the vitelline membrane of the oocytes.

The white follicles further grow to about 500 µm diameter still in groups of several follicles without clear hierarchical order and are summarized as small white follicles. The accumulation of cBuc, mainly produced from the granulosa cells, later found in the Balbiani body, and the increased intensity of CVH more actively synthesized in the cells with cBuc, proceeds continuously, and could be visualized as increasing intensity of labeling throughout the follicle development. Around the Balbiani body of follicles up to 300 µm (#) the condensed signals of cBuc (green, Fig. 4 e) are surrounded by a circular arrangement of for CVH (red, Fig. 4 f–h).

### Relative quantification of cBuc—mRNA expression in chicken follicles

The expression of cBuc in follicles < 100 µm, follicles between 100 and 500 µm as well as follicles of 500–1000 µm diameter was investigated with intron—spanning primers of the cBuc sequence applying rt-PCR as described earlier^32^.

**Fig. 8:**
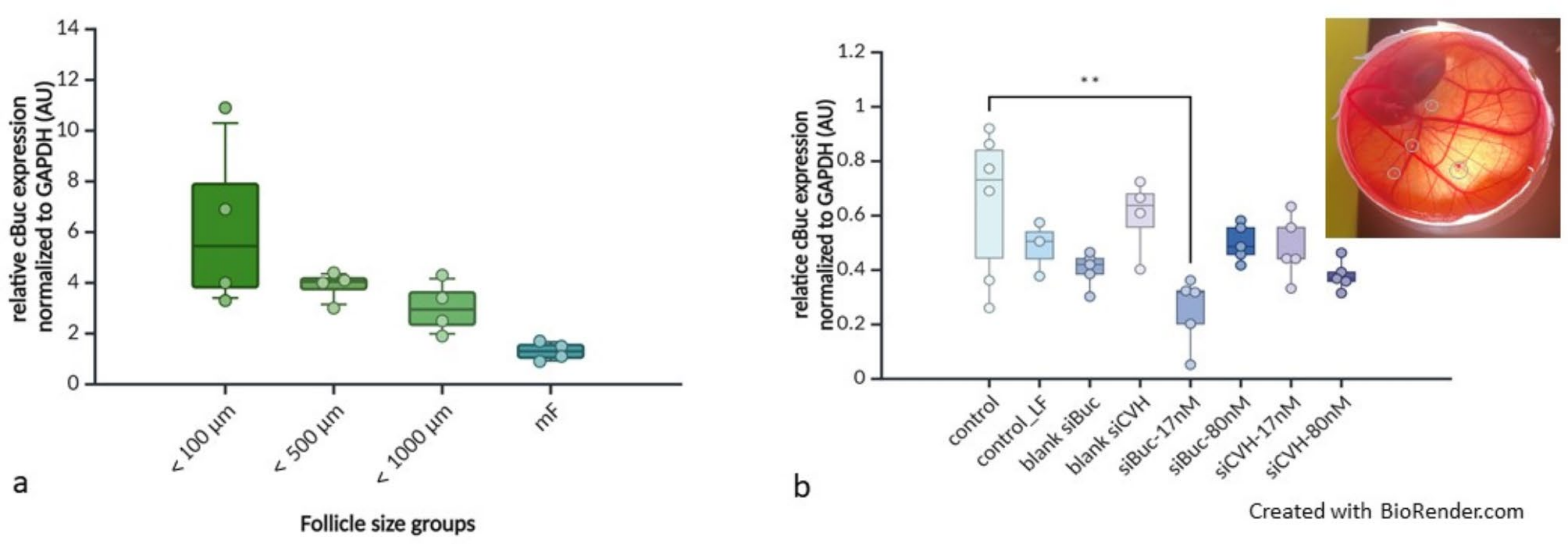
(**a**) Relative expression of cBuc in four follicle size groups and RNA interference. (**a**) Relative chicken Bucky ball abundance normalized to GAPDH. For rt-PCR of pools from 10 slowly growing white follicle fragments each for three follicle diameter groups and two triplets of germinal vesicles of freshly isolated maturing follicles were used. (mF = maturing follicles F5 to F3 and F1-2, Corresponding data and electrophoresis is shown in S-Figure S10. (**b**) RNA interference experiments in transplanted follicle cultures. Transcription of chicken Bucky ball was standardized to GAPDH of pools of 3 to 5 transplanted follicle fragments. After siRNA lipofection for 2 h, and co-incubation of the follicle fragments in the chorioallantoic membrane of E8 to E11 chicken embryos for additional 72 h (integrated fragments in the CAM are indicated by of embryo culture [grey circles] in the right upper corner image); the follicles < 100 µm indicated a significant lower transcription of Bucky ball after Bucky ball siRNA treatment at 17 nM after 72 h follicle co-culture. The treatment groups are labeled at the X-axis: control – medium control; control-LF—lipofectamine without siRNA; blank “X” – 80 nM siRNA for cBuc and CVH respectively without lipofectamine; siBuc-17 nM / 80 nM; siRNA for CVH—17 nM / 80 nM indicate siRNA treated follicle groups.

The highest relative mRNA expression for cBuc was recorded in the smallest follicles up to 500 µm diameter, and a lower cBuc—transcription continued to the maturing follicles as documented in Fig. 8a and Supplementary Fig. S9a and S9b. The larger growing follicles between 500 and 2000 µm diameter were characterized clearly by a reduced relative cBuc mRNA (Supplementary Figure S9a and S9b). In the maturing follicles the relative cBuc mRNA content was further reduced.

### RNA interference experiments with cBuc—and CVH – siRNA

The smaller dosage of 17 nM siBuc revealed a significant reduction of cBuc expression in the summary of two independent experiments and two and three independent RT-analyses of each experiment. No significant influence of interfering siRNA at doses of 80 nM for cBuc or either dosage of CVH during 2 h of lipofection at 37 °C could be recorded on the mRNA expression of cBuc for the follicles separated in the size range < 100 µm (Fig. 8 b, S-Fig. S10 a, S10 b). However, In the second group of follicles from 100 to 500 µm, a first experiment including these follicles had a similar trend of a reduction in cBuc, after the smaller cBuc-siRNA dosage. The highest dose of 80 nM siBuc did not result in a reduction in cBuc in this first experiment yet.

## Discussion

In this study, we demonstrate a sequential and dynamic accumulation and localization of cBuc around early chicken oocytes and in follicles, with an especially strong condensation of cBuc into the Balbiani body of primary follicles and de-condensation and re-location during later stages of growing follicles. In concert with an accumulation and condensation of cBuc in the Balbiani body of primary follicles, a specifically increased CVH concentration in the germ plasm attached to the granules of cBuc could be recorded. This dynamic pattern of cBuc localization and accumulation observed fits to the importance of Bucky ball in chicken as germ plasm organizer with the Balbiani body as structural unit for the germ plasm formation.

We investigated resting oocytes in the ovarian epithelium, small white to growing yellow yolk collecting follicles, and maturing follicles.

Follicles comprising continuously growing yolk size of 1 mm to 8 mm which are not yet ordered in a hierarchical sequence and referred to as pre-hierarchical follicles^46^. They are arranged on the surface of the ovaries as high numbers of small yellow follicles and had not been investigated in this study yet.

First cBuc positive cells were detected in the maternal ovarian stromal tissue around resting oocytes still in oocyte nests. Although the literature describes oocyte nests being degraded up to 35^th^ day after hatching^47^, we could repeatably find a few of these nests in ovaries from adult animals. However, within these resting oocytes, neither cBuc nor CVH could be detected yet.

In the ovarian epithelium however, most oocytes were found at least in stage II of primordial follicles released from these nests and at a minimum size of the oocytes of 50 µm with the beginning of the formation of the granulosa cell layer and a slow growth of oocytes. The granulosa cells directly surrounding the oocytes are at first still loosely arranged and rounded in the youngest follicles, but start synthesizing cBuc.

For the zebrafish oocyte development zBuc had not been reported in the somatic follicle cells yet, although Riemer et al. described the localization of zBuc in the Balbiani body in early stage 1b oocytes^48^.

However, the cBuc synthesis and transport into the oocytes before Balbiani body formation in primary oocytes is in agreement to the data reported by Heim et al.^49^ and Bontems et al.^20^, who demonstrated zBuc as specific regulator responsible for organizing oocyte polarity by formation of the Balbiani body and thereby germ plasm segregation. In chicken primary follicles with detectable Balbiani body, cBuc intensively labeled this structure and was characterized by successive condensation in discrete granules. The same development could be verified for *Xenopus* recently^25^.

The early polarizing effects for the Balbiani body in chicken oocytes is indicated by the eccentric localization of chromatin in close contact to the Balbiani body in primary follicles and confirmed the polarization of the oocyte via eccentrically shifting of the nucleus as reported early by Greenfield^50–53^. Similar polarization of the nucleus and Balbiani body is reported in zebrafish ^1^. Interestingly, in the current study an asynchronous distribution of stromal ovarian cells was observed that synthetize cBuc while the oocytes are still in the germ cell nests, and for the primordial and primary follicles.

The germ plasm organizing effect of cBuc in the current study is supported by the local increase of the germ plasm protein CVH. Although a weak, and uniform distribution of CVH could be visualized early on in stage II chicken follicles, increasing CVH concentrations were localized in close proximity to formed Balbiani structures of follicles already containing cBuc at follicle sizes of at least 90 µm diameter. This pattern fits to the functional data provided for zebrafish by Krishnakumar et al.^26^ and Pereira et al.^54^ who demonstrated direct binding of zebrafish Bucky ball and VASA and additionally the conserved binding of VASA by the sequentially unrelated but functionally congruent Oskar protein of *Drosophila* and a conserved aggregating functionality for VASA and DAZL as germ plasm proteins. The binding of Bucky ball and VASA in zebrafish could be verified to intensify VASA synthesis by enhanced zf-VASA ATPase activation^54^.

During further follicle development in chicken, the protein CVH^54^ is found perinuclear or within the nuclei of the oocytes separated from the accumulating CVH protein around the Balbiani body. Bucky ball was never seen in the nuclei of the oocytes in oocyte nests and in young follicles with the first arranging granulosa cells, but steadily in the growing granulosa cell layer with a one-sided fan of protein from the vitelline membrane directed to the Balbiani body, that could indicate continuous protein transport from the granulosa cells to the Balbiani body of the oocyte. The morphological maturation process of the granulosa cell layer during this early phase of primary follicle growth was measured as an increase in cell volume and stratification before by Marza^51,53^ and is reflected by an steadily increased localization of cBuc during granulosa layer maturation in this study.

The higher intensity of cBuc labeling in the granulosa cells on one side of the GV in all stages of the maturing follicle further reflect the polarizing effect of cBuc accumulation as it was reported in zebrafish^7,8,49^. This is in agreement to the synthesis of CVH in the oocyte itself reported by Guraya^55^, and further agrees to the pattern described for CVH by Tsunekawa et al.^38^ and DAZL by Lee et al.^56^ in the large maturing follicles. Both authors described for CVH as well as DAZL the ring-like protein accumulation at the GV of maturing follicles^57^ in chicken.

The data of dynamic cBuc protein synthesis in follicles was further investigated for mRNA expression. We recorded strong RNA levels for cBuc in small white primordial and primary follicles, whereas the relative mRNA content in maturing germinal vesicles was considerably lower. These data were recorded during the establishing phase of the experimental system and need verification via real time quantitative PCR.

However, the data fit to an early organizing function of cBuc as organizer for germ plasm proteins and RNA during oogenesis in a quite condensed state for the accumulation of germ plasm components. The immunoreactivity of cBuc independently demonstrates a first peak of cBuc protein with nuclear and perinuclear localization especially within the Balbiani body in primary follicles of stage III. The reduced relative RNA concentration in maturing oocytes correspond to the data recorded previously in mature oocytes and early embryogenesis where a second peak of protein was revealed during maternal to zygotic transition of expression in cleavage stages (EGK III to V) and nuclear localization of cBuc.^32^. These data correspond to the Bucky ball activity reported in zebrafish, where in the comparable stages of oocytes 1b and 2 Bucky ball acts as Balbiani body marker^48^.

The segregation of partially released germ plasm into a limited number of early cleavage cells is prepared by a partial de-condensation of the condensed Balbiani body into circular arranged germ plasm at the rim of the germinal vesicle. Thereby, germ plasm becomes prepared for the limited segregation into the first cleavage cells after fertilization.

This independently reflects the importance of the time of follicle formation for the organizing of the germ plasm by Bucky ball in chicken. First tests with interfering siRNA of Bucky ball resulted in an dosage dependent inhibiting effect on Bucky ball expression and provide a baseline for further experiments investigating the regulatory effect of Bucky ball on the collection of germ plasm components as reported to be conserved across vertebrate species^25^.

In summary, this study demonstrated the maternal origin and timeline for Bucky ball synthesis in early chicken folliculogenesis and in maturing follicles. The immunofluorescence data localizing Bucky ball in the Balbiani body and the intensified synthesis of the germ plasm protein CVH in close proximity to cBuc suggest a functional relationship as previously demonstrated in *zebrafish* oocytes. With the in-vitro follicle stimulation system established during this study, further experimental RNA interference experiments will help to elucidate the direct functional relationships between Bucky ball and germ plasm components.

## Materials and methods

### Generation of chicken Bucky ball monoclonal antibodies

#### Cloning and protein expression

A domain consisting of amino acids 155–354 of the chicken Bucky Ball (XP_040518588.1) was amplified from plasmid pT2RMCE_cBuckyball-Venus^32^ and subcloned in the pET15b-expression vector (Novagen, Merck KGaA, Darmstadt, Germany). The construct was fused to an N-terminal 6 × His-Tag and a C-terminal double StrepTag.

The pET15b-expression plasmid was transformed in BL21 (DE3) competent E. coli (#C25276H, New England Biolabs, Frankfurt am Main, Germany). For protein expression, cultures were grown in LB Lennox medium supplemented with 100 µg/ml ampicillin (#K029.4, Carl Roth, Karlsruhe, Germany and induced with 1 mM Isopropyl β- d-1-thiogalactopyranoside (#11IPTG0001, MP Biomedicals Germany, Eschwege, Germany) at an OD value of about 1.0. After incubation for 4 h at 37 °C, the cells were harvested by centrifugation. The pellets were resuspended with lysis buffer consisting of 100 mM Tris–HCl, 150 mM NaCl, 1% (v/v) Triton-X100 (#T8787, Sigma Aldrich Merck KGaA, Darmstadt, Germany); pH 8.0, supplemented with 1 mg/mL lysozyme (#8259.1, Carl Roth, Karlsruhe, Germany), 1 × protease inhibitor (#04693116001, Roche, Mannheim, Germany) and Benzonase (#E1014, Sigma Aldrich; Merck KGaA, Darmstadt, Germany). The lysates were incubated for 1 h at room temperature with shaking, subsequently sonicated for 1 min (200 W) and clarified by centrifugation at 15,000 × g for 30 min at 4 °C.

The supernatant was purified using Strep-Tactin®XT 4Flow® high capacity resin (#2–5030-010, iba lifesciences, Göttingen, Germany) according to the protocol of the manufacturer. The proteins were eluted with 50 mM biotin (in 100 mM Tris–HCl, 150 mM NaCl, pH 8.0). All protein containing fractions were pooled and subjected to a second round of purification using NiNTA agarose resin (#30210, Qiagen, Hilden, Germany) under native conditions as recommended by the manufacturer. Elution was performed with 250 mM imidazole. Finally, the pooled eluates were dialyzed overnight against an excess of 1 × PBS. Aliquots were stored at −80 °C until further use.

#### Generation of antigen-specific hybridoma cells

The immunization of mice was done in line with the general immunization program of the Friedrich-Loeffler-Institut (Landesamt für Landwirtschaft, Lebensmittelsicherheit und Fischerei, Mecklenburg-Vorpommern, permit: 7221.3–2-042/17).

Female BALB/c *mice* were immunized two times intraperitoneally and 5 times subcutaneously with 25 µg of recombinant Bucky ball expressed in *E. coli* strain BL21 (DE3) mixed with an equal amount of GERBU Adjuvant MM (GERBU Biotechnik GmbH, Germany) in intervals of 4 weeks. Four days after the final boost, the immunized mice were euthanized and the spleens were removed under aseptic conditions. Cell fusion and further subcloning steps were performed as described elsewhere^58^.

### Recovery of ovarian follicles

Adult hens of commercial layer hybrids or hens from genetic resource lines of the Friedrich-Loeffler-Institute with regular daily ovulations were used. Animals were maintained and handled according to the German laws regulating animal welfare and had been approved by the Animal welfare body of the Friedrich-Loeffler-Institut, Lower Saxony and the Lower Saxony State Office for Consumer Protection and Food safety. All hens originate of the institutional resources of the Friedrich-Loeffler-Institut before usage for organ retrieval.

The recovery of maturing follicles and ovaries with differently sized oocytes and resting oocytes was performed in agreement to the German and European animal rights legislation.

The German national legislation (Tierschutzgesetz $4, Abs. 3) does not require ethical approval for the humane killing of animals for the purpose of organ removal. However, the local Animal welfare body of the Friedrich-Loeffler-Institut approves all animal euthanasia procedures and checks that all methods have been carried out in accordance with the relevant guidelines and regulations. The killing of hens was performed after percussive blow to the head with a wooden stick and exsanguination after opening the A. carotis and V. jugularis with a knife.

From the ovary, the series of 5 to 8 maturing large yellow follicles, white growing follicles of 250–800 µm diameter, and ovary fragments including small resting, primordial, and primary follicles were recovered. The superficially accessible germinal disc regions (GD) from maturing yellow follicles were excised to remove the large yolk of up to 35 mm diameter. Oocytes within the ovary were differentiated by their diameter and coverage by granulosa and theca cell layers, as well as yolk accumulation. Finally, maturing follicles were staged by their yolk size hierarchically (Fig. S11, F1–F7). The vascularized theca layers were removed, and the GD excised as described before^32^.

### Fixation of follicles and germinal discs fragments, immuno-histochemistry

The fixation, immuno-histochemistry and imaging were performed as described before^32^. The explanted GD fragments as well as different sized oocytes in ovary fragments with resting oocytes of 30–800 µm were fixed in 4% formaldehyde (Carl-Roth, Karlsruhe, Germany) in 0.1 M PBS (Sigma-Aldrich, Taufkirchen, Germany) for about 4 h. Thereafter the samples were transferred in 0.1 M PBS + 0.1% NaN_3_ for a maximum of 14 d before immunostaining or stored frozen for cryo-sectioning after transfer in 30% sucrose.

Cryo-sections of 35 µm thickness were placed on poly-L-lysin coated slides for immuno-staining. The antibody labeling for Bucky ball and CVH was performed as described for cleavage embryos^32^. Shortly, selected slides with cryo-sections were washed 6 × in PBS, a 2 h-blocking step was performed in 0.02 M PBS including 0.05% Triton X100 and 1% bovine serum albumin (BSA). The primary antibodies were diluted in 0.02 M PBS, 0.1% BSA, 0.1% NaN_3_, 0.05% Triton X100.

The poly-clonal anti-cBuc were optimized at a 1:500 dilution of serum, and the hybridomas were optimized at 1:100 dilutions. All other antibodies and stains were applied as documented in Supplementary Table 1. After incubation for 44–48 h at 4 °C and several washes in PBS, the corresponding secondary antibodies were applied for 3 h. After washing, the sections were covered with Vectashield mounting medium including either SIR or DAPI as DNA-counterstain.

For validation of specificity of binding, each immunohistochemical procedure contained negative control samples (samples without primary antibodies and complete panel of secondary antibodies. Additionally, an isotype rabbit-IgG control was tested (Supplementary Table 1).

In a total of four different experiments, ovaries and uterine embryos from 10 to 12 hens each were recovered. From a set of 2 to 4 hens, the follicles of different stages, ova, and embryos recovered had been included in parallel in the same labeling experiments.

### Confocal imaging

Stacks of 25–30 µm were acquired from the differently sized oocyte stages and germinal disc region fragments including 20 to 25 single optical sections of 3 µm at uniform acquisition settings (12 bit images, 1024 dpi) at a confocal microscope LSM 510 using AIM software (Zeiss Microimaging Göttingen, Germany). Complete acquisition parameters are saved in the raw data database and are available upon request.

### Cell culture and transfection

DF-1 cells (CCLV-RIE 1029) were cultured in DMEM high glucose medium supplemented with 2 mM L-glutamine, non-essential amino acids, penicillin/streptomycin, (Capricorn Scientific, Ebsdorfergrund), 0.1 mM mercaptoethanol, 0.4 mM sodium pyruvate, 10% fetal calf serum, (ThermoFisher Scientific, Schwerte, Germany).

For transfections of the pT2RMCE-cBuckyball-Venus (with chicken Bucky ball cDNA)^32^ or the pCS2-buc-eGFP (zebrafish Bucky ball cDNA), a Biorad Pulser (Biorad, München, Germany) was used, essentially as described^59^ (2 square wave pulses, 300 V, 10 ms).

### Western blot analysis

Five days after transfection, the DF1 cells were washed and lysed in radioimmuno-precipitation buffer (RIPA, 500 µl per well of a 6-well plate) for 30 min on ice as described^60^. Pellet and soluble fractions were run on a 10% SDS-PAGE, and blotted to polyvinylidene difluoride (PVDF) membrane^32^. A mix of proteins containing IgG epitopes were used as size ladder (Blot I, SmoBio, #WM1000, Germany). After blocking with 5% non-fat milk powder, the PVDF membranes were probed with either sera samples from immunized mice (1:500), or hybridoma supernatants (1:250). Bound antibodies were detected via a horseradish peroxidase–coupled secondary anti mouse IgG antibody (1:10,000; Sigma) and an enhanced chemiluminescence reagent (Westar Supernova, Cynagen, #XLS3).

### RNA extraction and expression analysis

From pools of frozen follicles RNA extraction was performed of 4–6 fragments after cell lysis in Trizol with ZR bashing beads (S6014-50, Zymo Research) and Direct-zol RNA miniprep (R2052, Zymo Research, Freiburg, Germany) according to the protocol recommended by the provider. Reverse transcription was performed with supplemented DNase1 (RT-kit 4368814 applied biosystems, USA) from 500 µg RNA and PCR-conditions and primers as reported previously^32^. Quantifications were performed on PCR after fixed cycles and intensity quantification of electrophoresis band intensities with the software Gelanalyzer.

### RNA experiments with lipofection of small interfering oligonucleotides for cBuc and CVH

Mixed groups of follicle fragments were separated from ovaries of two adult reproductive active hens and selected to contain 3–6 follicles each at a size up to 100 µm. A total of 60 of these fragments were pooled for each treatment group and washed twice in PBS and cooled in OptiMEM before transfection. Lipofection was performed with lipofectamine 3000™ (ThermoFisher scientific, USA) and siRNAs for cBuc and CVH as recommended in the manual of the producer. Control groups were included as medium control, treated with lipofectamine alone, siRNA co-incubation without lipofectamine for both genes. Two doses of 17 nM and 80 nM siRNA were applied for each siRNA sequence in two independent experiments. The co-incubation was performed in 4 drops with 15 fragments each in a total volume of 100 µl for 2 h at 37 °C and 5% CO_2_.

### *In-ovo* co-culture of follicle fragments on the chorioallantoic membrane of chicken embryos

After completed lipofection, 4 to 5 fragments containing the follicles were transplanted within the chorioallantoic membrane of embryonic day 8 chicken embryos which had been cultured from the third day of incubation within surrogate host shells as described by Perry in the third phase of culture^61^. After 24 h of co-incubation, successfully integrated transplants in the chorioallantoic membrane were recognizable by neovascularization. After 72 h of co-incubation these follicle fragments were harvested and frozen at 82 °C for further analyses.

### Statistical analysis

The treatment effects of siRNA were analyzed from relative RNA expression values as quotients of cBuc to GAPDH expression calculated with Gelanalyzer software from 2% agarose gel grey values. The statistical analysis was performed from 5 different PCR analyses including two siRNA experiments with two and three independent reverse transcriptions of RNA from 4–6 pooled samples for each treatment. One-way ANOVA with Dunnett multiple comparison tests was performed with R4.22 (Fig. S13) in the software ‘Biorender’.

## Supplementary Information

Supplementary Information 1.

Supplementary Information 2.

## Data Availability

Upon request the databases with the imaging raw data will be provided by the corresponding author.

## Abbreviations

cBuc: Chicken Bucky ball
zBuc: Zebrafish Bucky ball
zBuc-pAB: Polyclonal antibodies directed against the *zebrafish* Bucky ball
CDH: Dead End Homologue
CVH: Chicken vasa homolog
DAZL: Deleted in Azoospermia Like
DND: Dead end
GD: Germinal disc
GV: Germinal vesicle
IDP: Intrinsically disordered proteins
LLPS: Liquid–liquid phase separation
TDRDn: Tudor domain containing proteins
PGCs: Primordial germ cells
PVDF: Polyvinylidene difluoride membrane
siBuc: Small interfering RNA oligo for *cBuc*
siCVH: Small interfering RNA oligo for *CVH*
