# Supplementary material for: Dynamic maternal synthesis and segregation of the germ plasm organizer, Bucky ball, in chicken oocytes and follicles
Source: Sci Rep. 2024 Nov 12;14:27753. doi: 10.1038/s41598-024-78544-7 (PMC11557578; doi:10.1038/s41598-024-78544-7)
Supplement: Supplementary file 1 — Supplementary Information 1. [file 41598_2024_78544_MOESM1_ESM.pdf]

Dynamic maternal synthesis and segregation of the germ plasm organizer, Bucky ball, in chicken oocytes and follicles

Sabine Klein[#1], Roland Dosch[2], Sven Reiche[3], Wilfried A. Kues[1]

Supplementary file titles:

Supplementary Table S1: Antibodies and stains

Supplementary Figure S1: Comparison of immunolabeling for the three cBuc hybridomas (8B2, 7G4, 5B3) with positive immunoreactivity

Supplementary Figure S2: Direct comparison of cBuc-monoclonal and zBuc-polyclonal antibody labeling in primary (stage III) and growing follicles (stage IV)

Supplementary Figure S3: Controls for immunostaining without primary antibodies and isotype control

Supplementary Figure S4: Folliculogenesis in the chicken ovary

Supplementary Figure S5: Co-labeling with cross-reactive polyclonal antibody against zBuc (green) and CVH (red) in follicles of stage I & II

Supplementary Figure S6: Primary follicle of stage III with Balbiani body (#) and attached cell nucleus (N)

Supplementary Figure S7: Identification of the oocyte´s nucleus (N) without antibody treatment aside the Balbiani body

Supplementary Figure 8: Co-labeling with cross-reactive polyclonal antibody against zBuc and CVH in maturing follicles (stage V)

Supplementary Figure 9a: Electrophoresis and Gelanalyzer data for relative expression of cBuc- and GAPDH-RNA in follicles of different size classes

Supplementary Figure 9b: Electrophoresis and Gelanalyzer data for relative expression of cBuc- and Polymerase A - RNA in follicles of different size classes

Supplementary Figure 10a: Statistics report – ANOVA details
Report of the statistical analysis of relative cBuc expression normalized to GAPDH for small white follicles at a size limit of 100 µm diameter from two RNA interference experiments. The box plot graph is presented in Fig. 8b.

Supplementary Figure 10b: Statistics report continued from 10a; test for normality of data distribution and variance equality check details
