# Supplementary material for: Dynamic maternal synthesis and segregation of the germ plasm organizer, Bucky ball, in chicken oocytes and follicles
Source: Sci Rep. 2024 Nov 12;14:27753. doi: 10.1038/s41598-024-78544-7 (PMC11557578; doi:10.1038/s41598-024-78544-7)
Supplement: Supplementary file 2 — Supplementary Information 2. [file 41598_2024_78544_MOESM2_ESM.pdf]

Supplementary Table 1: Antibodies and stains

	Provider	ID	concentration
**MitoBrillant 646**	Tocris	7700	100 nM
**Phalloidin_Alexa555**	Life technologies	A34055	20 nM
**DAPI**	SIGMA	D9542	1 µg/ml
**SIR**	Spirochrome	SC007	2 µM
**AlexaFUOR 488**	Life technologies	A-11059	1 µg/ml
**AlexaFLUOR 555F(AB)**	Life technologies	A21430	2 µg/ml
**Mouse IgG1 Isotype control**	Biozol	ROC-010-001-331	4 µg/ml
**VASA antibody**	Gene tex	HL2485	5 µg/ml
**zf-Buc (gp-IgG, rb-IgG)**	Gift Roland Dosch	--	1 µg/ml
**Vectashield mounting medium**	Vector	H1000	na

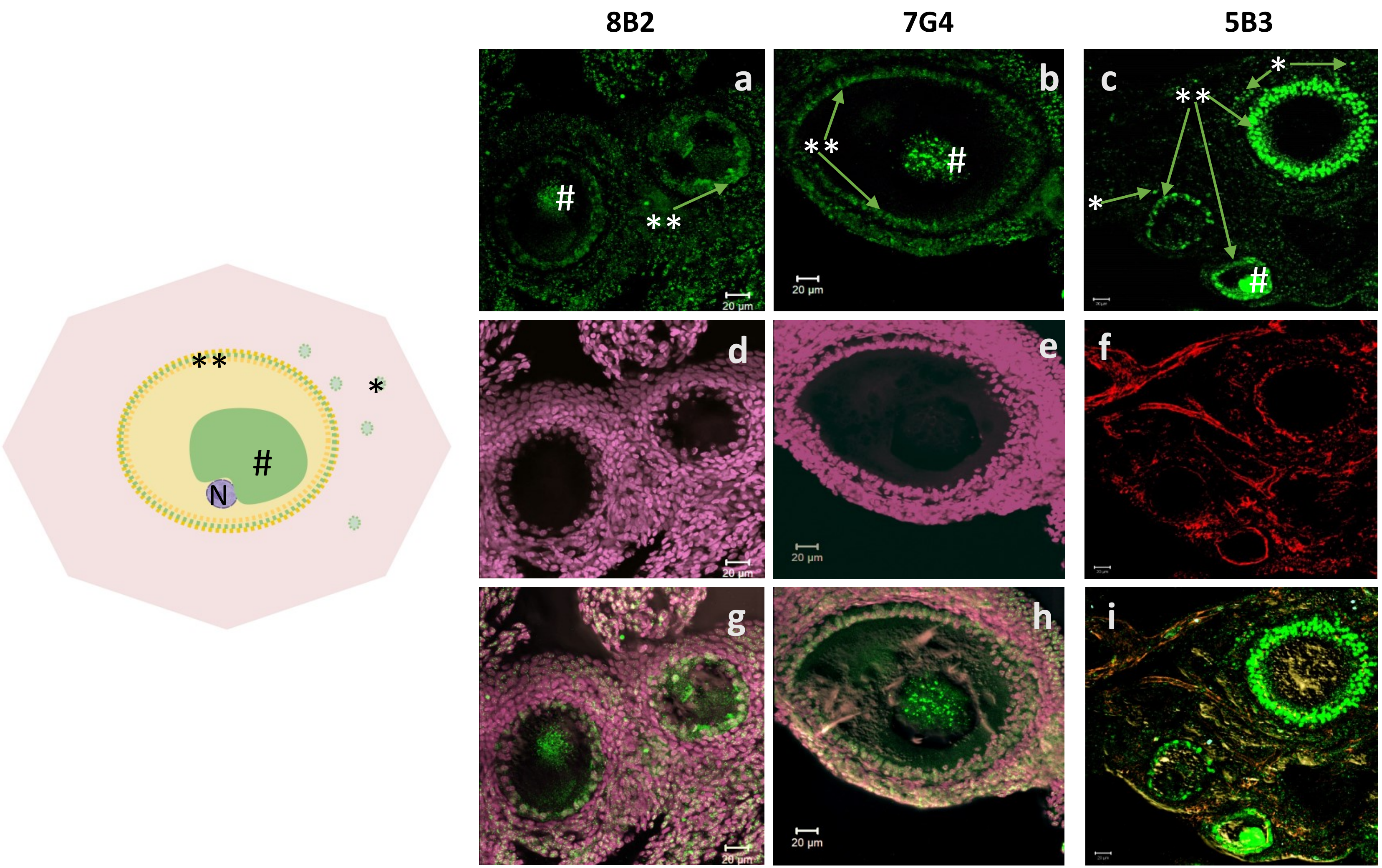

Supplementary Figure S1: Comparison of immunolabeling for the three cBuc hybridomas (8B2, 7G4, 5B3)

Examples of primary follicles (stage III, follicles 80 – 150 µm, 35 µm sections) are presented, demonstrating cBuc labeling of Balbiani bodies (#) and granulosa cells (**); column 1: hybridoma 8B2, column 2: hybridoma 7G4, column 3: hybridoma 5B3, Frame a-c: cBuc label in green; There is higher stromal background labeling in hybridomas 8B2 and 7G4. Only few stromal cells are labeled with 5B3 (*). For hybridomas 8B2 and 7G4 (column 1 and 2 respectively) the nuclei are labeled with SIR (frames d, e magenta), In column 3 (hybridoma 5B3) phalloidin was applied as independent label for actin filaments (f, red). The bottom row (g-i) provides the overlay for the cBuc and SIR/phalloidin labels accomplished by DIC in each column. Scales: 20 µm

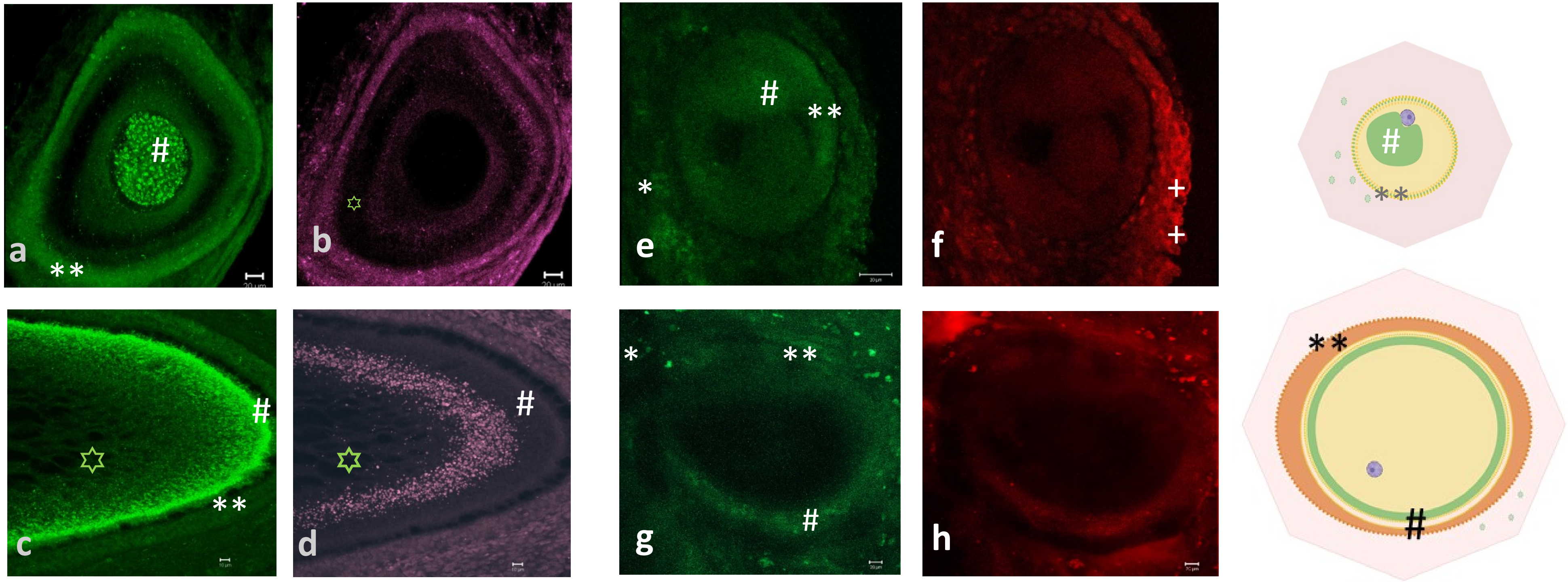

Supplementary Figure S2: Direct comparison of cBuc-monoclonal (a-d) and zBuc-polyclonal (e-h) antibody labeling in primary (stage III) and growing follicles (stage IV)

The cBuc monoclonal labeling (a,c) is more intense and visualizes the granular condensation of cBuc especially in the Balbiani body (a, stage III) and the de-condensation in the semi-circular attachment of cBuc in growing follicles (c, stage IV). The SIR counterstaining (b, d, ✡) visualizes RNAs aside the cBuc localization in both stages. The polyclonal zBuc antibodies did not resolve the granular condensation in the Balbiani body (#, e) at much weaker signal intensity, however the co-localization to CVH within the follicle, especially with the Balbiani body was already apparent (g and h).

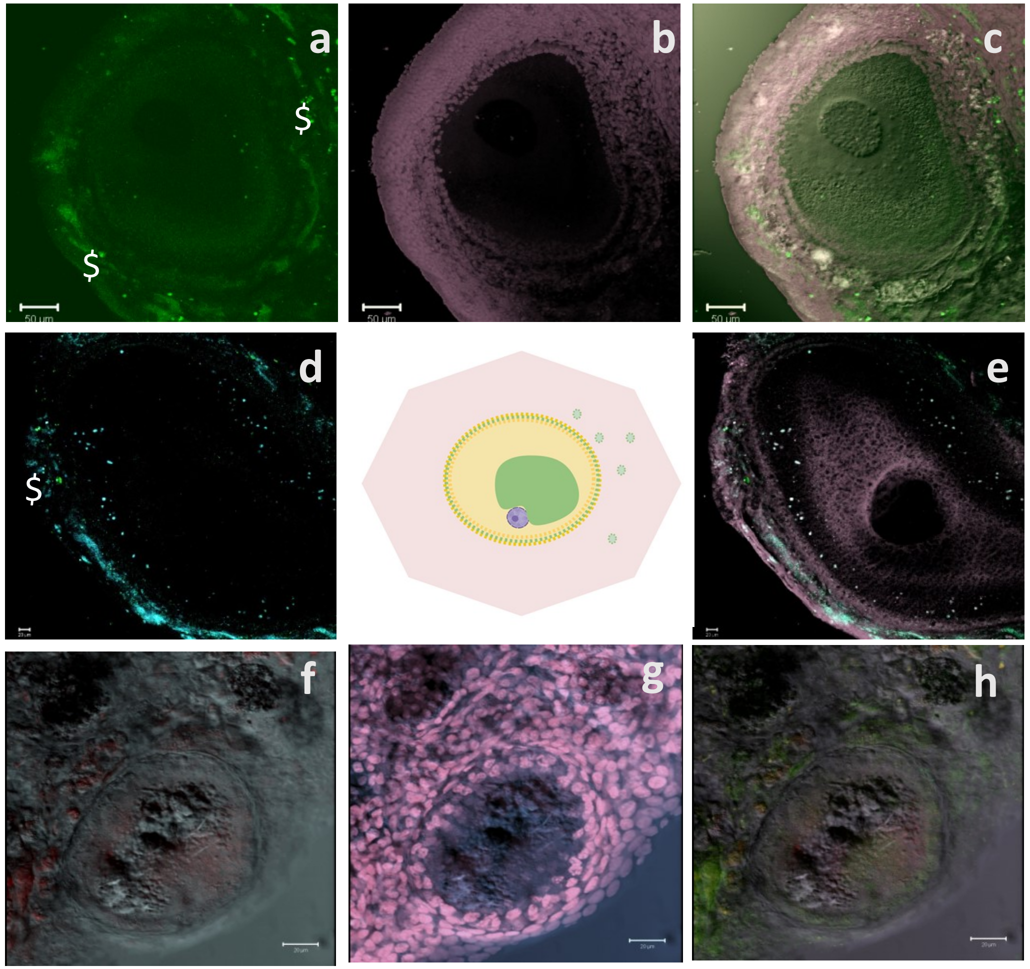

Supplementary Figure S3: Controls for immunostaining without primary antibodies and isotype control

Two control samples treated in antibody solution without primary antibodies, but complete set of secondary antibodies (a-e) and isotype control with α-mouse IgG1 as primary antibody and the same set of secondary antibodies (f-h). In the upper panel SIR was used as DNA-stain (b and c magenta); in the middle panel (d and e) phalloidin (cyan in d) and Mitobrillant-red - magenta) were applied in combination with Bucky ball (green). In d only green fluorescent spots are detectable as caused of yolk particles. In the lower panel (f-h) a mouse IgG1 antibody (red in f) was applied in combination with an anti-mouse Alexa 555 (red in f) and SIR (g magenta) and anti-rabbit Alexa 488 secondary antibody (h green),
All these controls do not show fluorescence as observed with the zBuc- and cBuc antibodies within the follicles scales a-c: 50 µm, scales d-h: 20 µm

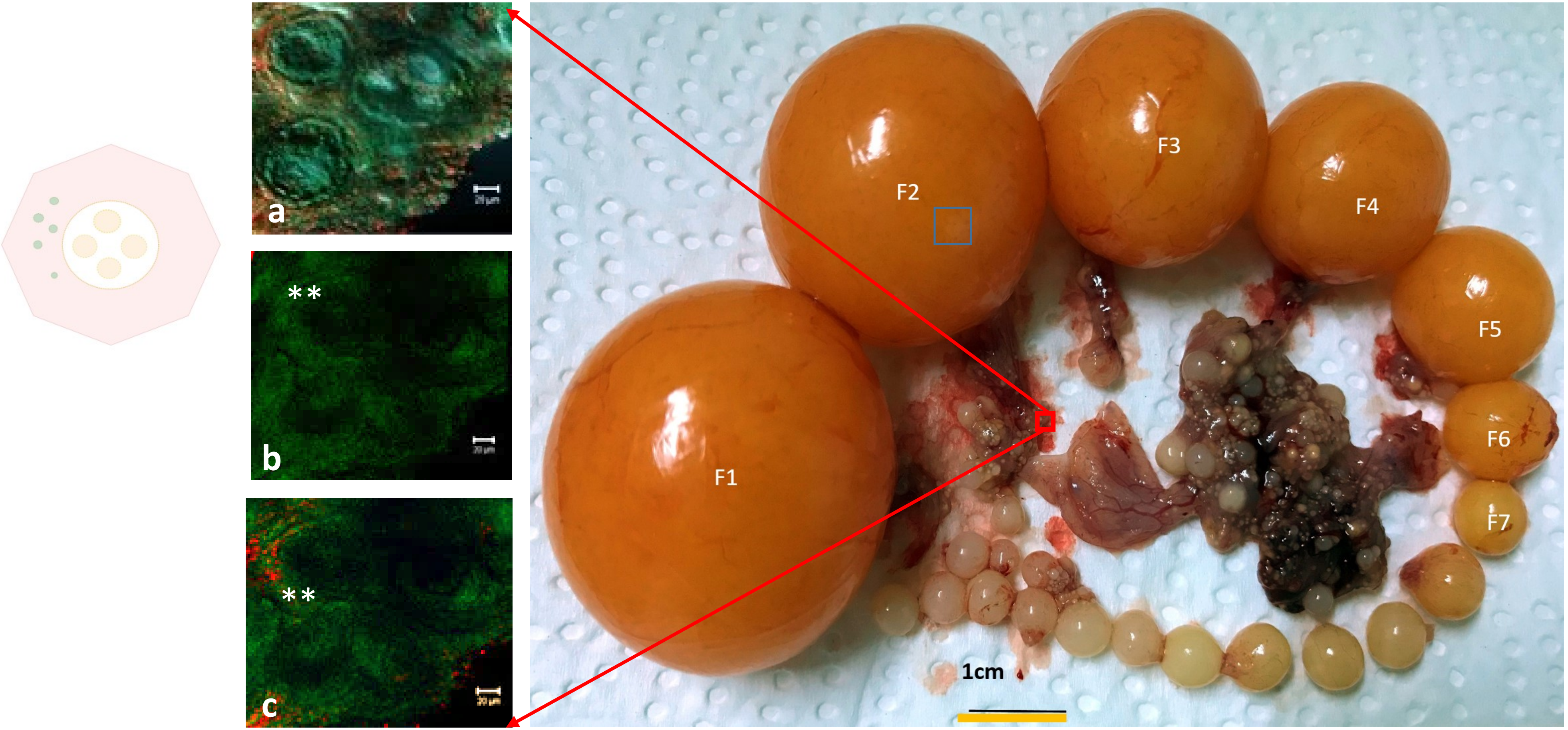

Supplementary Figure S4: Folliculogenesis in the chicken ovary

Right side – explanted chicken ovary with maturing follicles hierarchically arranged according to their yolk size and patches of small yellow and white primary follicles, Left side: Scheme for stage I of chicken oogenesis with one oocyte nest including four oocytes and surrounded by stromal ovarian tissue with single cells containing cBuc (green) and immunostaining of an red rectangular frame of ovarian epithelium including small primary follicles <60 µm , scales: 20 µm

a: overlay of stained for DNA with DAPI – cyan, DAPI + cBuc + CVH, DIC, b: cBuc – green with first labeling of granulosa cells (**), c: overlay of cBuc (green) +CVH (red)

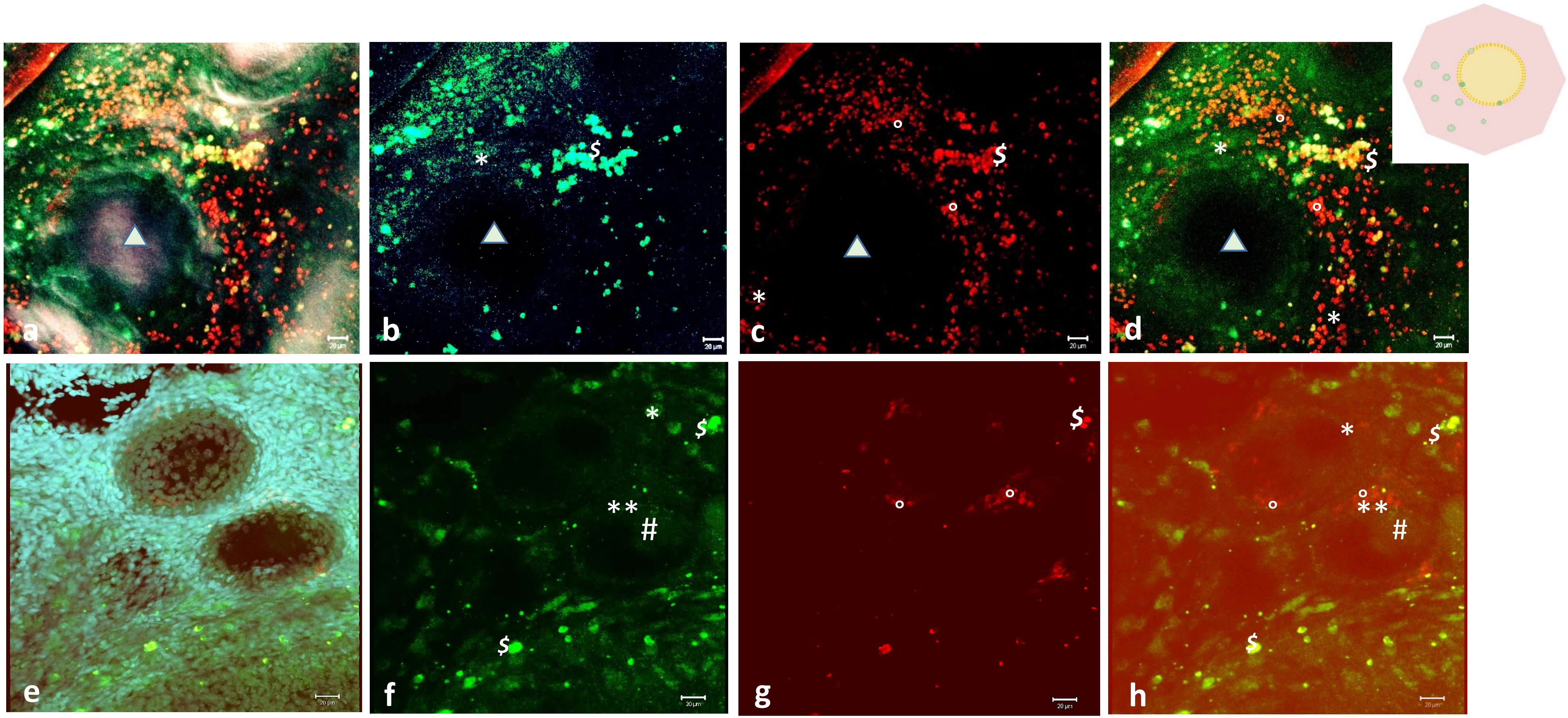

Supplementary Figure S5: Co-labeling with cross-reactive polyclonal antibody against zBuc (green) and CVH (red) in follicles of stage I & II

Two examples demonstrating follicles of stage I (a-d) and stage II (e-h); Stromal ovarian cells between primary follicles at a follicle size of around 50 µm are labeled independently for CVH (˚ red, in c, g) and cBuc (*, green, b, f). An oocyte which is not yet labeled is marked by a triangle in a - d; Larger spots positive for both labels are indicated by $ and originate probably from yolk particle autofluorescence also apparent in controls without antibody against Bucky ball as shown in (Supplementary Figure S3a - h). An # indicates first accumulation of cBuc within the oocyte in f and h. scales: 20 µm

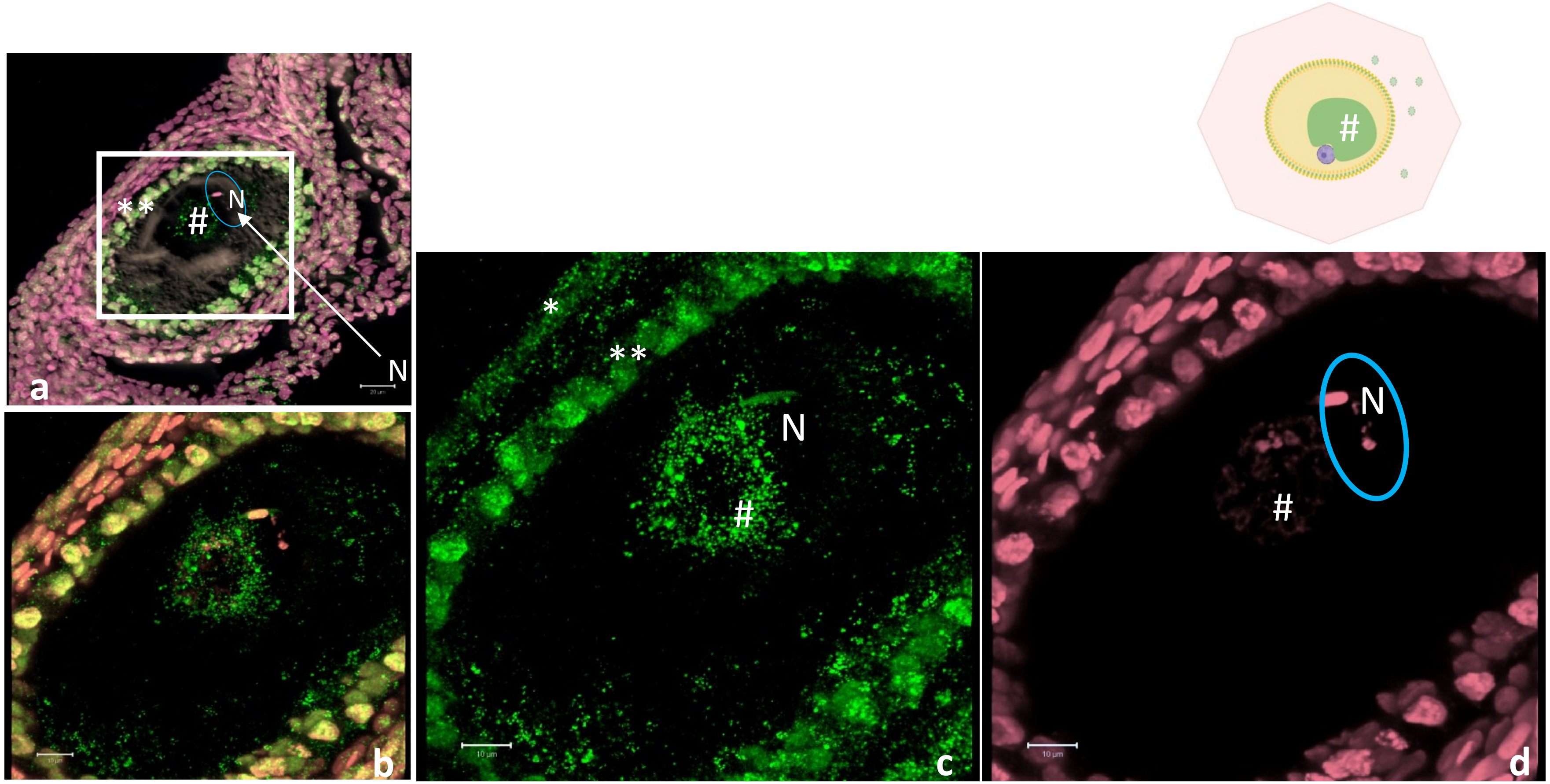

Supplementary Figure S6: Primary follicle of stage III with Balbiani body (#) and attached cell nucleus (N)

This follicle (130 µm follicle diameter) visualizes the eccentric position of the nucleus (N) within the primary follicle with the close attachment of the Balbiani body (#) containing the granular condensed cBuc (green) and more diffuse cBuc staining of the granulosa cells (**). The blue demarcation outlines the nucleus, barely visible in the overlay with the DIC contrast image in frame a, scale: 20 µm; Nucleoli of the oocyte nucleus are clearly stained with SIR (magenta spots within the blue demarcation in a) and could be confirmed in the magnified frame centered around the Balbiani body (b + d). Frame a provides the overview of the follicle as overlay with cBuc (green) and SIR (magenta) staining, frame c visualizes the cBuc label and frame d shows SIR label separately. scales: 10 µm (b-d)

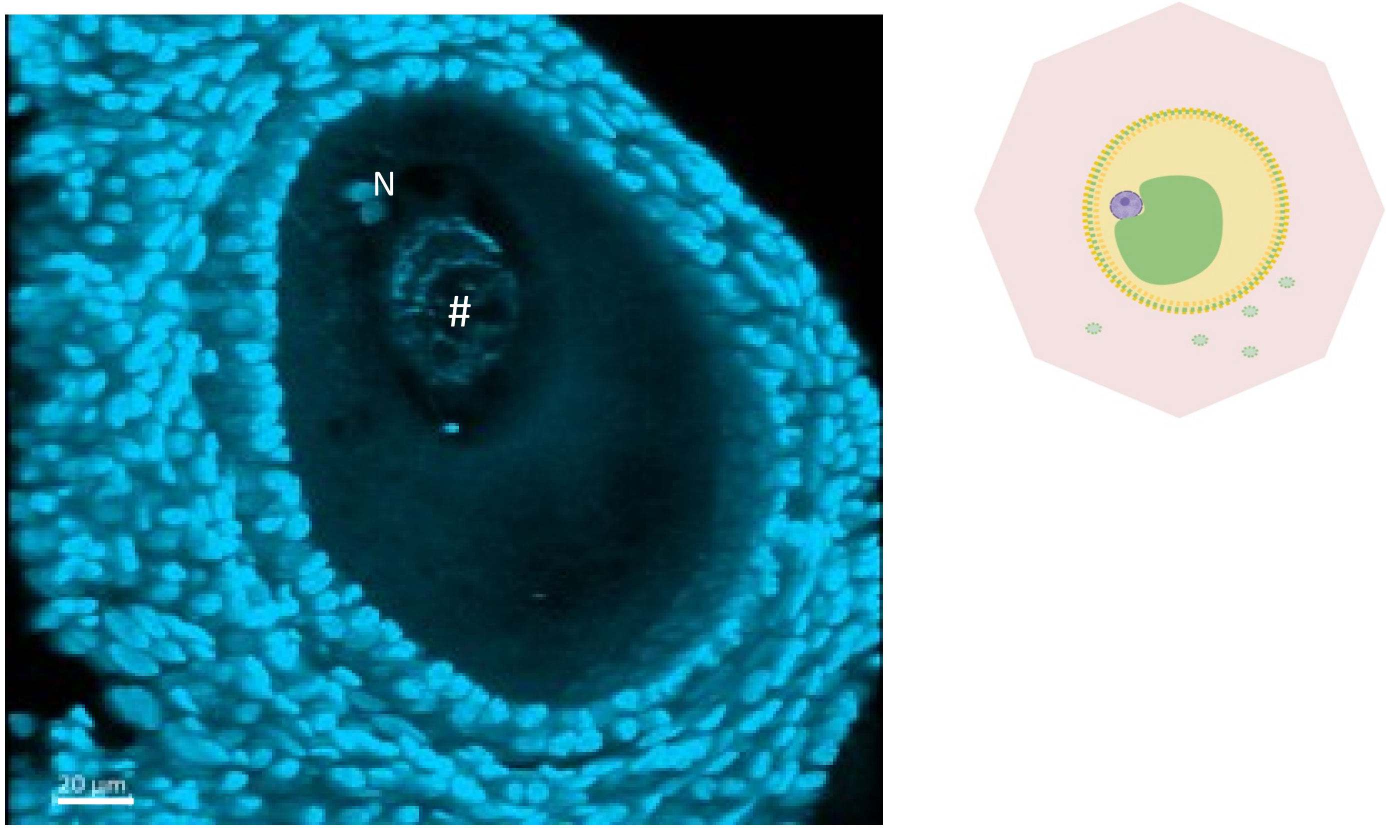

Supplementary Figure S7: Identification of the oocyte´s nucleus without antibody treatment aside the Balbiani body
DAPI stained nucleic acids in a primary follicle of about 150 µm diameter. Two nucleoli of the nucleus (N) are stained aside the fussy RNA-label within the Balbiani body structure (#). The oocyte is surrounded by the densely packed nuclei of the granulosa cells and stromal tissue, scale: 20 µm

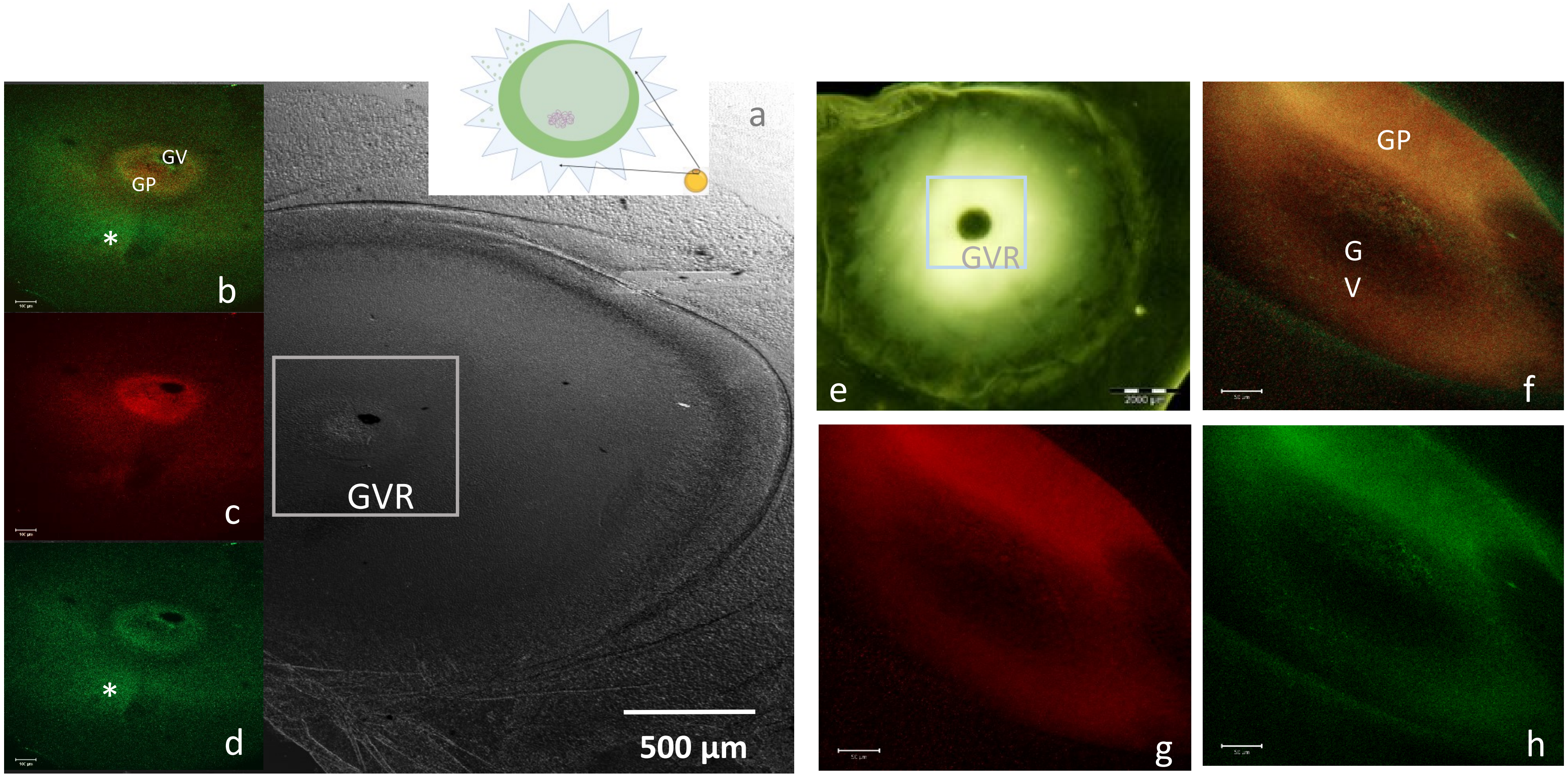

Supplementary Figure S8: Co-labeling with cross-reactive polyclonal antibody against zBuc and CVH in maturing follicles

Germinal vesicle region of two F1 follicles labeled for c-Buc (green) and CVH (red) indicating eccentric concentration of the germ plasm containing cBuc and CVH predominantly at one side of the geminal vesicle ; I-IV first germinal vesicle region, the rectangle from the DIC overview in I demonstrates in the germinal vesicle region circular enrichment of Bucky ball and CVH around the vesicle with higher concentration at one side of the germinal vesicle, the overlay of cBuc and CVH in II, CVH alone in III, and cBuc label in IV, additionally to the circular labeling of cBuc around the germinal vesicle, an additional cell group is labeled for cBuc exclusively aside the vesicle in the surrounding follicular cells (IV *).

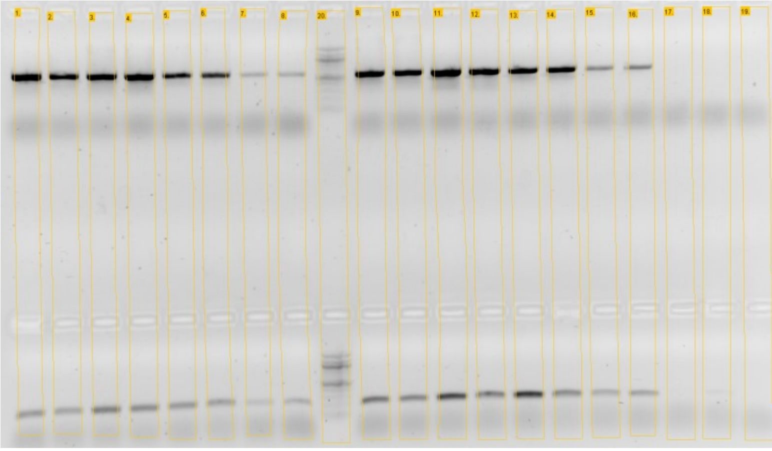

lane	Buc2	GAPDH	Buc/GAPDH	mean Buc/GAPDH
1 / H1_AA1	3051	439	6.9	7.9
2 / H1_AA2	1702	161	10.6	
3 / H1_BB1	2719	675	4.0	4.2
4 / H1_BB2	2584	587	4.4	
5 / H1_CC1	1556	453	3.4	2.9
6 / H1_CC2	1132	459	2.5	
7 / F3-5_1	354	209	1.7	1.1
8 / F3-5_2	412	484	0.9	
20/ molecular weight ladder NEB 3231				
9 / H2_AA1	2636	799	3.3	3.6
10 / H2_AA2	2518	632	*4.0*	
11 / H2_BB1	3172	1045	3.0	3.4
12 / H2_BB2	2796	690	4.1	
13 / H2_CC1	2134	1150	1.9	2.6
14 / H2_CC2	2293	538	4.3	
15 / F1_2-1	515	464	1.1	1.3
16 / F1_2-2	565	385	1.5	
17 / MM_cDNA1		na		
18 / MM-cDNA2		na		
19 / MM_cDNA3		na		

S9a

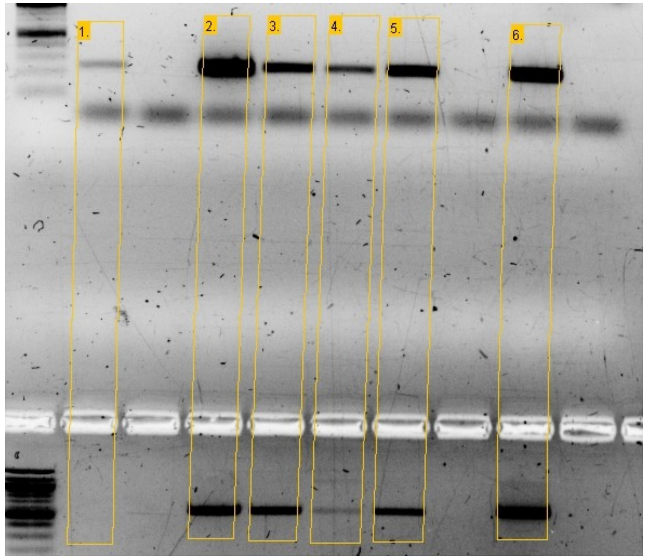

	Controls AA < 100 µm, CC < 500 µm			
lane	Vol_PolyA	Vol_Buc	Buc/PolymA	follicle type
	molecular weight ladder NEB 3231			mean
testis	460	0	0,0	
A-AA	4354	1663	0,4	AA = 0.50
B-AA	2250	1283	0,6	
A-CC	1283	196	0,2	CC = 0.25
B-CC	2949	1014	0,3	
RT--				
C-AA	3132	1689	0,5	
MM				

S9b

Supplementary Figure 9a:

Electrophoresis and Gelanalyzer data for relative expression of cBuc- and GAPDH-RNA in follicles of different size classes

Gel documentation of a transcription experiment for chicken Bucky ball- and GAPDH- mRNA from total RNA- extracts of pools from 10 slowly growing white follicle fragments each for three follicle size groups and two triplets of germinal vesicles of freshly isolated maturing follicles RNA extracts from two adult laying hens, mF=maturing follicles F5 to F3 and F1-2, respectively. MM-cDNA (n) refers to controls of independent reverse transcription set ups without RNA input. The statistical analysis is represented as box plot in Figure 9a.

Supplementary Figure 9b:

Electrophoresis and Gelanalyzer data for relative expression of cBuc- and Polymerase A - RNA in follicles of different size classes

Gel documentation of a transcription analysis for chicken Bucky ball- and Polymerase A - mRNA from total RNA - extracts of pools from 6 follicle fragments each of two different size groups of follicles: AA - smaller than 100 µm diameter and CC - follicles larger than 500 µm diameter

One-way ANOVA

All statistical tests are run using R 4.2.2. in the software BioRender

One-way ANOVA summary

F, DFn, DFd	3.9030, 7.0000, 30.0000
P-value	0.004 **
Eta squared (η^2)	0.477

One-way ANOVA additional details

	SS (sum of squares)	DF	MS (mean of squares)
Between columns	0.5582	7	0.07974
Within columns	0.6128	30	0.02043
Total	11710.0510	37	

Descriptive statistics

Category	Minimum	25th percentile	Median	75th percentile	Maximum
control	0.258	0.3345	0.729	0.8745	0.918
control_LF	0.375	0.375	0.503	0.572	0.572
blank siBuc	0.3	0.3415	0.418	0.4515	0.463
blank siCVH	0.4	0.4518	0.635	0.7073	0.722
siBuc-17nM	0.05	0.125	0.316	0.3405	**0.36**
siBuc-80nM	0.415	0.435	0.484	0.5665	0.58
siCVH-17nM	0.33	0.3845	0.44	0.5925	0.631
siCVH-80nM	0.313	0.3345	0.366	0.4255	0.461

Category	Mean	SD	SEM
control	0.6423	0.2717	0.1109
control_LF	0.4833	0.09996	0.05771
blank siBuc	0.4008	0.063510	0.02844
blank siCVH	0.598	0.1401	0.07005
siBuc-17nM	0.2494	0.1265	0.05657
siBuc-80nM	0.4974	0.06834	0.03056
siCVH-17nM	0.4788	0.1162	0.05199
siCVH-80nM	0.3772	0.05450	0.02437

Supplementary Figure 10a: Statistics report – ANOVA details

Report of the statistical analysis of relative cBuc expression normalized to GAPDH for small white follicles at a size limit of 100 µm from two RNA interference experiments. The box plot graph is presented in Fig. 8b.

Sapiro-Wilk normality test

Category	W	p-value		Passed normality test (alpha=0.05)?	n
control	0.8861	0.2983	ns	Yes	6
control_LF	0.97010	0.67210	ns	Yes	3
blank siBuc	0.9214	0.5389	ns	Yes	5
blank siCVH	0.9031	0.4469	ns	Yes	4
siBuc-17nM	0.8647	0.2458	ns	Yes	5
siBuc-80nM	0.9514	0.7474	ns	Yes	5
siCVH-17nM	0.9602	0.8093	ns	Yes	5
siCVH-80nM	0.9558	0.7786	ns	Yes	5

Levene test of equal variances

F	DFn	DFd	p-value	Are the SDs significantly different?
1.7169	7.0000	30.0000	0.1428 ns	No

Supplementary Figure 10b: Statistics report continued

Test for Normality of data distribution and variance equality check details
